# Multilocus genotype analysis outlines distinct histories for *Trichinella britovi* in the neighboring Mediterranean islands of Corsica and Sardinia

**DOI:** 10.1186/s13071-018-2939-9

**Published:** 2018-06-19

**Authors:** Giuseppe La Rosa, Isabelle Vallée, Gianluca Marucci, François Casabianca, Ennio Bandino, Fabio Galati, Pascal Boireau, Edoardo Pozio

**Affiliations:** 1 European Union Reference Laboratory for Parasites, Istituto Superiore di Sanità, Rome, Italy; 2 Animal Health Laboratory, OIE Collaborating Centre for Foodborne Zoonotic Parasites, JRU BIPAR, Anses, ENVA, INRA, Université Paris Est, Maisons-Alfort, France; 3 INRA, Corte, France; 4 Istituto Zooprofilattico Sperimentale della Sardegna, Nuoro, Italy

**Keywords:** *Trichinella britovi*, Molecular epidemiology, Mediterranean islands, Pig, Wild boar, Fox, Trichinellosis, Microsatellite, Pork products

## Abstract

**Background:**

The zoonotic nematode *Trichinella britovi* was discovered in two neighboring Mediterranean islands of Corsica and Sardinia, almost simultaneously at the beginning of the 21st century. An epidemiological link between the two parasite populations was generally assumed. In 2015, an outbreak of trichinellosis in Nice, the South of France, was reportedly caused by the consumption of raw pork delicatessen imported from Corsica. The aims of the present study were to investigate, by multilocus genotype (MLG) analyses, the hypothesis of the common origin of the Corsican and Sardinian *T. britovi* foci and to trace “from fork to farm” the origin of the pork product, which caused a trichinellosis outbreak in mainland France in 2015.

**Methods:**

Sixty-three *T. britovi* isolates were collected from animals and pork products of Sardinia and Corsica islands and from mainland of Italy, France and Spain. We analyzed genetic variability at four polymorphic microsatellite loci by two independent algorithms, the Bayesian and multivariate analyses, to evaluate the genetic relationships of 1367 single larvae.

**Results:**

*Trichinella britovi* isolates of the two islands showed different genetic structures and the Bayesian analysis revealed a different membership of the two insular populations. Furthermore, two geographically separate genetic groups were identified among Corsican isolates. Lastly, the origin of the pork delicatessen marketed in Nice was linked to a breeder-butcher in Corsica.

**Conclusions:**

The low level of genetic admixture of the insular *T. britovi* isolates suggests that this pathogen colonized the two islands by separate events. On the other hand in Corsica, although the isolates share the same genetic structure, geographically separate isolates showed different membership. We suggest the MLG analysis as a suitable method in supporting epidemiological investigations to trace “from fork to farm” insular populations of *T. britovi*.

**Electronic supplementary material:**

The online version of this article (10.1186/s13071-018-2939-9) contains supplementary material, which is available to authorized users.

## Background

Parasites of the genus *Trichinella* are zoonotic nematodes circulating among wild carnivore and omnivore animals with a cosmopolitan distribution on all continents except Antarctica [1]. When humans fail to properly manage domestic pigs and wildlife, *Trichinella* spp. are transmitted from the sylvatic to the domestic environment, triggering the onset of the domestic cycle [2]. Farming practices at risk of *Trichinella* spp. transmission occur, in general, in disadvantaged and poor areas due to several reasons including the lack of veterinary services, difficulties in controlling the myriad of small pig units, the rearing of pigs in backyards as well as the practice of allowing pigs to roam freely in the wild without any feed control [2].

Except for Sicily, where *Trichinella spiralis* was documented in pigs and humans from 1933 to 1961 [3], *Trichinella* spp. have never been recorded from the islands of the Mediterranean Basin until 2004, when *Trichinella britovi* was detected in free-ranging pigs in a remote mountainous area of Corsica [4]. One year later, a human outbreak of trichinellosis occurred in neighboring Sardinia following the consumption of pork from a free-ranging pig reared in a remote area of the island [5]. In the following years, extensive surveys showed that *T. britovi* was circulating among free-ranging pigs and wildlife of the two islands and an epidemiological link between the Corsican and Sardinian parasite populations was suspected due the almost simultaneous detection of the parasites on both islands, their geographical proximity, and the illegal animal trade between the two regions [6–8]. In 2015, an outbreak of trichinellosis occurred in the region of Nice, the South of France, due to the consumption of raw sausages imported from Corsica [9].

The Bayesian and multidimensional analyses of multilocus genotype data may constitute a useful tool to study the genetic structure of *Trichinella* populations originating from different continents and from restricted areas [10–12].

The aims of the present study were to investigate, by multilocus genotype analyses, the hypothesis of the common origin of the Corsican and Sardinian *T. britovi* foci and to trace “from fork to farm” the origin of the pork product, which caused a trichinellosis outbreak in mainland France in 2015. The results reject the hypothesis that *T. britovi* shares a common history in Corsica and Sardinia, and trace back the pork origin of the trichinellosis outbreak of Nice to a Corsican village.

## Methods

### Investigated areas

Corsica (France) with a surface area of 8680 km^2^ is the most mountainous island in the Mediterranean Sea. Mountains make up two-thirds of the island rising 2700 m in height with deep and steep valleys. Almost 20% of the island is forest, and 3500 km^2^ of the territory is preserved as a nature reserve. The human population density of 37 inhabitants per km^2^ is about 1/3 that of continental France.

Sardinia (Italy) is the second-largest island in the Mediterranean Sea, with an area of 24,100 km^2^. A sea loch of only 11 km separates this island from Corsica. Mountains cover about 13.6%, hills about 67.9% and plains about 18.5% of the surface. The human population density of 69 inhabitants per km^2^ is about 1/3 that of continental Italy.

### *Trichinella* spp. isolates

In the present work, we conceive the terms: (i) “individual larva” or just “larva” as a single nematode organism of the genus *Trichinella* collected from striated muscles of naturally infected animals or meat products by artificial digestion; (ii) “isolate” as a group of larvae collected from striated muscles of naturally infected animals or meat products, by artificial digestion; and (iii) “population” as the *T. britovi* isolates present in each of five investigated areas, namely the Mediterranean islands of Corsica and Sardinia, and continental Italy, France and Spain (see below).

**Fig. 1:**
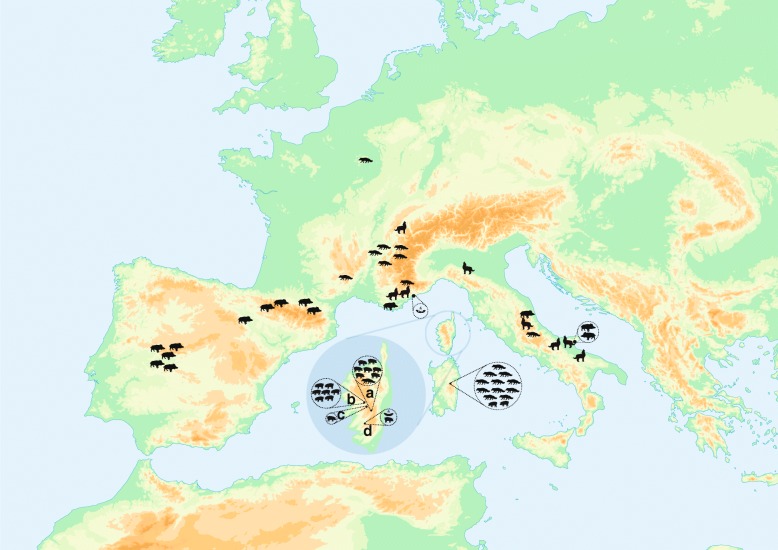
Geographical and host origin of *Trichinella britovi* isolates. Isolates investigated by microsatellite analysis originated from the Mediterranean islands of Corsica (*Key*: a, Cozzano; b, Vallée de Gravona; c, Bastelica; d, Aullène), Sardinia, and from the continent (Italy, France and Spain). Each silhouette shows a *T. britovi* isolate from domestic pig, wild boar, wolf, fox, or pork sausage

*Trichinella britovi* larvae were collected by artificial digestion according to the European Commission’s regulations [13, 14] from animals originating from the only known focus in Sardinia (Orgosolo, 13 isolates) and from four foci in Corsica (Cozzano, Vallée de Gravona, Aullène and Bastelica; 17 isolates) as well as from raw pork delicatessen, 1 figatelli produced in Aullène (Corsica) and marketed in the Nice area (continental France) and 1 sausage collected from the breeder-butcher of Aullène, where figatelli had been produced (Table 1, Fig. 1). For the comparison of the genetic structure of *T. britovi* populations of the two islands with those of continental Europe, *T. britovi* larvae were collected from animals from continental Italy (9 isolates), continental France (14 isolates) and continental Spain (8 isolates) (Table 1, Fig. 1). Single larvae were washed 4 times with distilled water on ice and stored in 5 μl of 90% ethyl alcohol at -20 °C.

**Table 1:** Main features of *Trichinella britovi* isolates used for microsatellite analysis

No.	ISS code^a^	Host/source	Locality of origin (region)	Country
1	1615	Domestic pig	Orgosolo (Sardinia)	Italy
2	4137	Red fox	Orgosolo (Sardinia)	Italy
3	4138	Red fox	Orgosolo (Sardinia)	Italy
4	3991	Red fox	Orgosolo (Sardinia)	Italy
5	4151	Red fox	Orgosolo (Sardinia)	Italy
6	4152	Red fox	Orgosolo (Sardinia)	Italy
7	4153	Red fox	Orgosolo (Sardinia)	Italy
8	4154	Red fox	Orgosolo (Sardinia)	Italy
9	4155	Red fox	Orgosolo (Sardinia)	Italy
10	4156	Red fox	Orgosolo (Sardinia)	Italy
11	4551	Domestic pig	Orgosolo (Sardinia)	Italy
12	4552	Red fox	Orgosolo (Sardinia)	Italy
13	6226	Red fox	Orgosolo (Sardinia)	Italy
14	1497	Domestic pig	Cozzano (Corsica)	France
15	1572	Red fox	Cozzano (Corsica)	France
16	1573	Domestic pig	Cozzano (Corsica)	France
17	1574	Domestic pig	Cozzano (Corsica)	France
18	1575	Domestic pig	Cozzano (Corsica)	France
19	1576	Domestic pig	Cozzano (Corsica)	France
20	1577	Domestic pig	Cozzano (Corsica)	France
21	1578	Domestic pig	Cozzano (Corsica)	France
22	4126	Domestic pig	Vallée de Gravona (Corsica)	France
23	4127	Domestic pig	Vallée de Gravona (Corsica)	France
24	4130	Domestic pig	Vallée de Gravona (Corsica)	France
25	4131	Domestic pig	Vallée de Gravona (Corsica)	France
26	4132	Domestic pig	Vallée de Gravona (Corsica)	France
27	4244	Domestic pig	Vallée de Gravona (Corsica)	France
28	4245	Domestic pig	Vallée de Gravona (Corsica)	France
29	4669	Domestic pig	Bastelica (Corsica)	France
30	nc^b^	Figatelli^c^	Aullène (Corsica)	France
31	nc^b^	Sausage^d^	Aullène (Corsica)	France
32	6303	Domestic pig	Aullène (Corsica)	France
33	5627	Wolf	Berceto (Emilia Romagna)	Italy
34	5591	Wolf	Andria (Apulia)	Italy
35	6340	Wild boar	Arquata del Tronto (Marche)	Italy
36	5610	Wolf	Monte S. Angelo (Apulia)	Italy
37	5637	Wolf	Teramo (Abruzzo)	Italy
38	6159	Red fox	Villavallelonga (Abruzzo)	Italy
39	6161	Wolf	Tufara (Molise)	Italy
40	nc^b^	Wild boar	Monte S. Angelo (Apulia)	Italy
41	nc^b^	Wild boar	Monte S. Angelo (Apulia)	Italy
42	244	Red fox	(Isère)	France
43	325	Red fox	Saint Pierre d'Allevard (Isère)	France
44	326	Red fox	Chichilianne (Isère)	France
45	327	Red fox	Entraigues (Provence)	France
46	348	Red fox	Entemont le vieux (Haute Savoie)	France
47	351	Red fox	Chignin (Haute Savoie)	France
48	352	Red fox	Rimaucourt (Marne)	France
49	137	Red fox	(Iozère)	France
50	1728	Wild boar	(Var)	France
51	2473	Wolf	(Var)	France
52	2474	Wild boar	(Ariège)	France
53	3992	Wild boar	(Ariège)	France
54	4418	Wolf	La combe du lars (Haute Savoie)	France
55	6302	Wolf	Péone (Alpes Maritimes)	France
56	255	Wild boar	Jarandilla (Extremadura)	Spain
57	5691	Wild boar	Deleitosa (Extremadura)	Spain
58	5694	Wild boar	Tornavacas (Extremadura)	Spain
59	5700	Wild boar	Fresnedoso de Ibor (Extremadura)	Spain
60	5703	Wild boar	Boltana (Aragon)	Spain
61	5710	Wild boar	Hontanares (Castile and León)	Spain
62	5716	Wild boar	Beraton (Castile and León)	Spain
63	5728	Wild boar	Sabinanigo (Aragon)	Spain

^a^Longitude and latitude values of the locality of isolate origin are available on the website of the International Trichinella Reference Center (https://trichinella.iss.it/) using these codes

^b^nc, no ISS code

^c^Figatelli originating from domestic pigs of Aullène village marketed in the Nice region (the South of France), where they caused a trichinellosis outbreak [9]

^d^The sausage was collected from the breeder-butcher, where the figatelli was produced

Since four species of *Trichinella* occur in Europe and may co-infect the same host [1], each larva was identified by multiplex PCR to exclude from the analysis individual larvae of either *T. spiralis*, *Trichinella nativa* or *Trichinella pseudospiralis* [15].

### Microsatellite analysis

Total DNA was purified from single larvae as previously described [11]. Single larvae were individually genotyped through the screening of four polymorphic microsatellite loci (Table 2). TS1010B and TS1380 microsatellites were previously described [11], whereas TB922 and TB1019 microsatellites were used here for the first time. PCR conditions for all the four markers are as previously described by La Rosa et al. [12].

**Table 2:** *Trichinella britovi* microsatellite primer pairs

Code	Length (bp)	Primer pairs (5'-3')	
		Forward	Reverse
TS1010B	240	CATTAACGATGTGCTATTTAACGCT	CCAACAACATCCTCAACA
TS1380	285	TCAATTCATTTCATTTCAATCTGCG	CACCTTACAATCAAGTAACC
TB1019	285	CGGACAGATTCAGCGGA	AGCCAACTCAAGTCCCAAA
TB922	250	ATGGGCCAACAACTACCACTA	AAACGGCAATGCAACAAC

Genotyping was accomplished by capillary electrophoresis of PCR products using the Qiaxcel device (Qiagen GmbH, Hilden, Germany) [11]. Alleles were coded by their size in nucleotide base pairs estimated by comparing each peak to reference peaks, whose size had been established by sequencing the amplified homozygote products. If novel alleles were amplified as homozygotes, they were sequenced and included as reference size.

### Genetic variability

Genetic variability per locus, isolate, population and overall loci, was assessed by computing: (i) the mean number of alleles per locus (Na); (ii) effective number of alleles (Ne); (iii) proportion of polymorphic loci (%Pl); (iv) observed heterozygosity (Ho); and (v) unbiased expected heterozygosity (He) under Hardy-Weinberg expectations (HWE). All values were assessed using Genepop v.4.3 software [16].

The deviation of genotypic frequencies from panmixia was evaluated according to HWE. The accordance to panmixia (as null hypothesis), for each locus per isolate, was evaluated by Fis using the exact test (nominal level *P* < 0.05) [17, 18] by Genepop v.4.3 (settings for Markov chain parameters: dememorization = 1000, batches = 100 and Markov chain Monte Carlo iterations per batch = 5000). The deviation of Ho from He was tested under the hypotheses of heterozygote deficiency or excess for overall loci per isolate (nominal level *P* < 0.05) by FSTAT v.2.9.3 [19]. Indicative adjusted nominal level to 0.0002 (5040 randomizations), after Bonferroni correction, was also probed by FSTAT v.2.9.3. Since no significant value was detected, these results are not shown. The Bonferroni correction was taken into account testing global HWE for all loci, for overall isolates of each of the five areas. The pairwise differentiation of allele frequencies among isolates and among areas, was tested by Fst and its significance (*P* ≤ 0.05) was computed by Arlequin v.3.5.2.2 [20].

Previous studies on microsatellite analysis of *T. spiralis* isolates have shown that the genetic structure of the isolates are likely unrelated since this parasite showed the capacity to accomplish transmission as highly inbred, admixed lineages and a mixture of both [12]. Consequently, multiple comparisons of the genetic differentiation within each investigated area, were carried out using average values instead of a global analysis.

### Genetic structure

The Bayesian clustering algorithm implemented in STRUCTURE v.2.3.3 [21, 22] was used to infer the genetic structure and relationships among multilocus genotypes (MLGs) of individual larvae. The estimated membership coefficients (*Q*) were evaluated for each larva and graphically represented by colors. An individual larva is characterized by a vertical bar, where each color represents the proportion of *Q* that assigns it to the same color inferred K cluster.

Multiple runs, assuming 1 to 10 subdivisions (K), were performed using a ‘burn-in’ of 200,000 followed by 100,000 Markov Chain Monte Carlo iterations. Ten simulations were carried out for each K, assuming “admixture” as the ancestry model and “independent frequencies” as allele frequency model. Bayesian analysis was used to perform a detailed evaluation of the genetic structure of the Corsican isolates. Realistic values for K were evaluated according to Evanno et al. [23] using pophelper webapps [24]. The genetic relationships among isolates were further evaluated by principal coordinates analysis (PCoA), which is unrelated to the Bayesian algorithm, since it does not rely on HWE. PCoA was performed by GenAlEx v.6.2 software [25].

## Results

### Genetic variability and differentiation

An average of 21.7 individual larvae (SE 0.23) from each of the 63 *T. britovi* isolates from Corsica, Sardinia, and continental Italy, France and Spain, were subjected to PCR amplification using four microsatellite loci. Successful amplification was achieved > 96% of the time (TS1010B = 99.7%; TS1380 = 97.5%; TB1019 = 96.5; and TB922 = 91.7%). No mixed infection with other *Trichinella* species was detected.

The microsatellite analysis of the 63 isolates revealed appreciable levels of genetic variability as shown by Na = 2.655 (SE 0.071), Ne = 1.868 (SE 0.044) and Ho = 0.373 (SE 0.014) (see Additional file 1: Table S1). All isolates showed at least 50% of polymorphic loci (Additional file 1: Table S1). Forty-one isolates were polymorphic at all four loci (%Pl = 100), 11 at three loci (%Pl = 75), and 11 at two loci (%Pl = 50) (Additional file 1: Table S1). Fis values of each locus per isolate showed a large variability ranging from negative (-0.429) to positive (0.832) values showing a significant larger (per 18 times) or lower (per 2 times) departure from expectations (nominal level for HWE exact test *P* ≤ 0.05).

In multiple comparisons, overall the four loci per isolate, the departure (nominal level *P* ≤ 0.05) of Ho from He as measured by Fis index, was detected for ten isolates (Nos. 12, 17, 18, 19, 22, 38, 47, 52, 56 and 58) (Additional file 1: Table S1). Nine of them displayed significant larger Fis values than expected, while isolate No. 56 showed a significant lower Fis value. The global test of HWE for all loci and all isolates within the five areas, showed a significant departure of *P* after Bonferroni adjustment, for all areas excluding Sardinia.

**Fig. 2:**
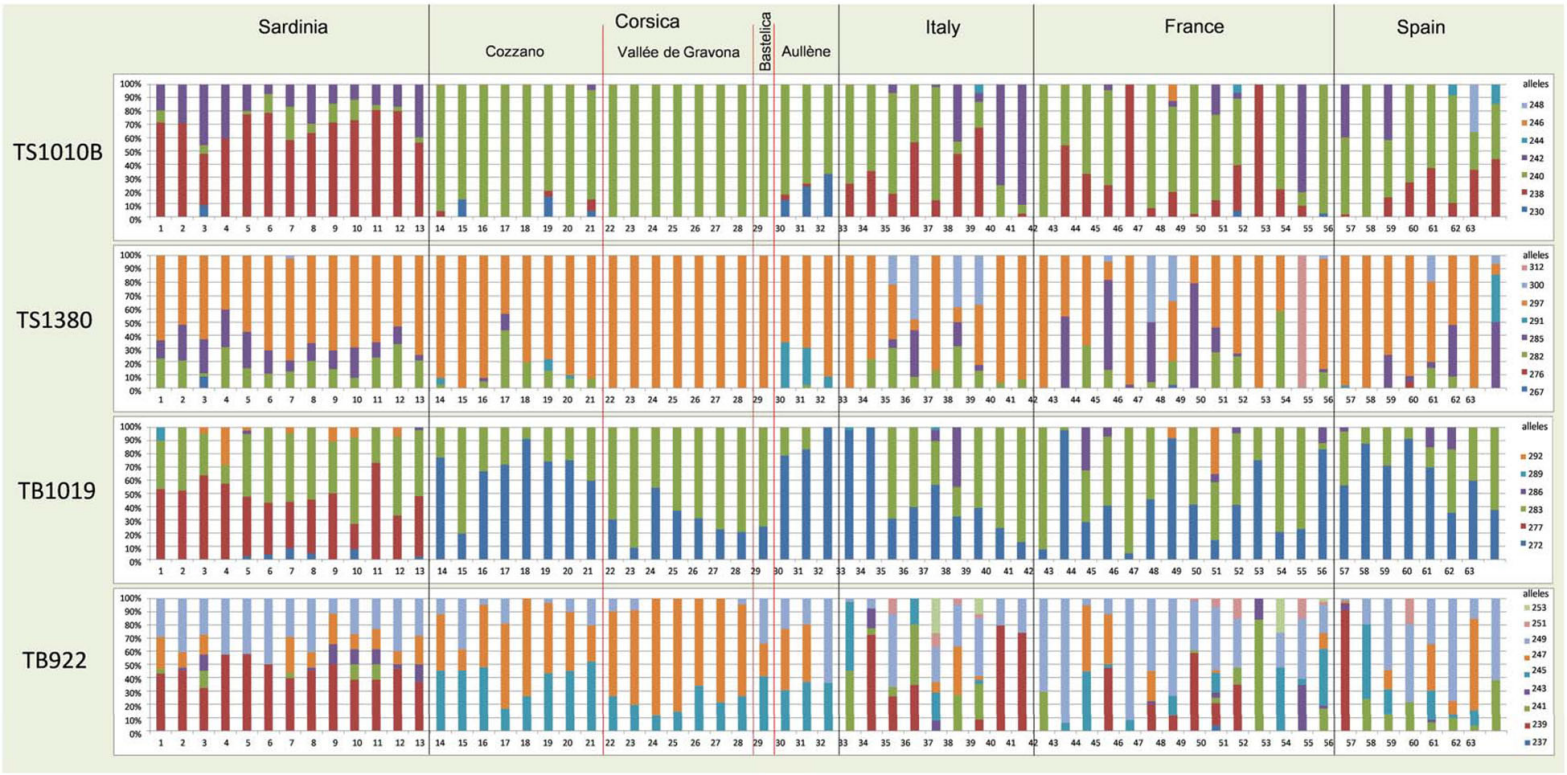
Allele frequencies of *Trichinella britovi* larval cohorts. Parasites derived from 63 animals (domestic pigs, wild boars, wolves, red foxes and meat products) of Corsica (four localities) and Sardinia islands, and of continental Italy, France and Spain (see Table 1 for isolate numbers)

Each geographical area was characterized by different level of genetic variability as suggested by Na and Ho values. The Corsican isolates showed the lowest average Ho value (0.251, SD 0.084) and the lowest number (*n* = 13) of alleles. No private allele (i.e. an allele detected in only one studied area) was detected among Corsican isolates. As shown by the Bayesian and PCoA analyses, the Corsican isolates were separated into two clusters, the one of Vallée de Gravona (Nos. 22–28) and the one of Cozzano (Nos. 14–21), which displayed a different genetic variability according to the average Ho values (0.170 *vs* 0.281) and allele number (*n* = 11 *vs n* = 13, Fig. 2; Additional file 2: Table S2). The Sardinian isolates showed the highest levels of genetic variability (average Ho = 0.527, SD 0.061), and the presence of 20 alleles, of which one private (TB1019/277) was detected in all the 13 isolates (Fig. 2, Additional file 2: Table S2).

*Trichinella britovi* isolates from continental Italy, France, and Spain, showed different levels of genetic variability with average Ho values of 0.409 (SD 0.149; 20 alleles), 0.357 (SD 0.170; 25 alleles), and 0.399 (SD 0.120; 21 alleles), respectively. *Trichinella britovi* from continental France was characterized by three private alleles, TS1010B/246, TB922/237 and TS1380/312, detected in isolate No. 48 (frequency 12%), No. 50 (frequency 4%) and No. 54 (frequency 100%), respectively. *Trichinella britovi* from continental Spain had two private alleles, TS1380/276 and TS1010B/248, detected in isolate No. 59 (frequency 4%) and No. 62 (frequency 36%), respectively. *Trichinella britovi* isolates from continental Italy did not show any private allele (Fig. 2, Additional file 2: Table S2). The low genetic variability of *T. britovi* in Corsica was further suggested by the highest number of fixed alleles (29%), whereas Sardinia and continental Italy, France and Spain showed 0, 5, 11 and 9% of fixed loci, respectively (Additional file 2: Table S2).

The analysis of the genetic differentiation among the 63 isolates (1953 pairwise estimates) evaluated by Fst, showed a significant departure (*P* ≤ 0.05) from the null hypothesis (i.e. all individuals belong to the same population) in 94.9% (1854/1953) of pairwise comparisons (Additional file 3: Table S3).

The analysis of the genetic differentiation within each investigated area, showed high average Fst values (± SD) (Corsica, 0.219 ± 0.184; continental Italy 0.348 ± 0.176; continental France 0.432 ± 0.215; and continental Spain 0.217 ± 0.118), except for Sardinia (0.031 ± 0.048) (Table 3).

**Table 3:** Average pairwise Fst values (SD) of *Trichinella britovi* isolates originating from Sardinia, Corsica and continental Italy, France and Spain

Investigated areas (no. of tested isolates)	Sardinia	Corsica			Continental		
		All foci	Cozzano	Vallée de Gravona	Italy	France	Spain
Sardinia (13)	0.031 (0.048)						
Corsica all foci (19)	0.498 (0.077)	0.219 (0.184)					
Cozzano (8)	0.279	nd^a^	0.069				
Vallée de Gravona (7)	0.310	nd^a^	0.122	0.036			
Continental Italy (9)	0.342 (0.123)	0.389 (0.181)	0.219	0.277	0.348 (0.176)		
Continental France (14)	0.396 (0.132)	0.415 (0.221)	0.230	0.297	0.391 (0.193)	0.432 (0.215)	
Continental Spain (8)	0.356 (0.124)	0.281 (0.148)	0.150	0.235	0.286 (0.166)	0.340 (0.199)	0.217 (0.118)

^a^nd, not done because Cozzano and Vallée de Gravona Fst values are already included in “All foci”

The analysis of the genetic differentiation among the investigated areas showed high average Fst values for all pairwise comparisons (Table 3). The highest average Fst value (0.498 ± 0.077) was detected between the two islands, while the lowest average Fst value (0.281 ± 0.148) was recorded between Corsica and continental Spain (Table 3).

### Bayesian analysis of *T. britovi* isolates

**Fig. 3:**
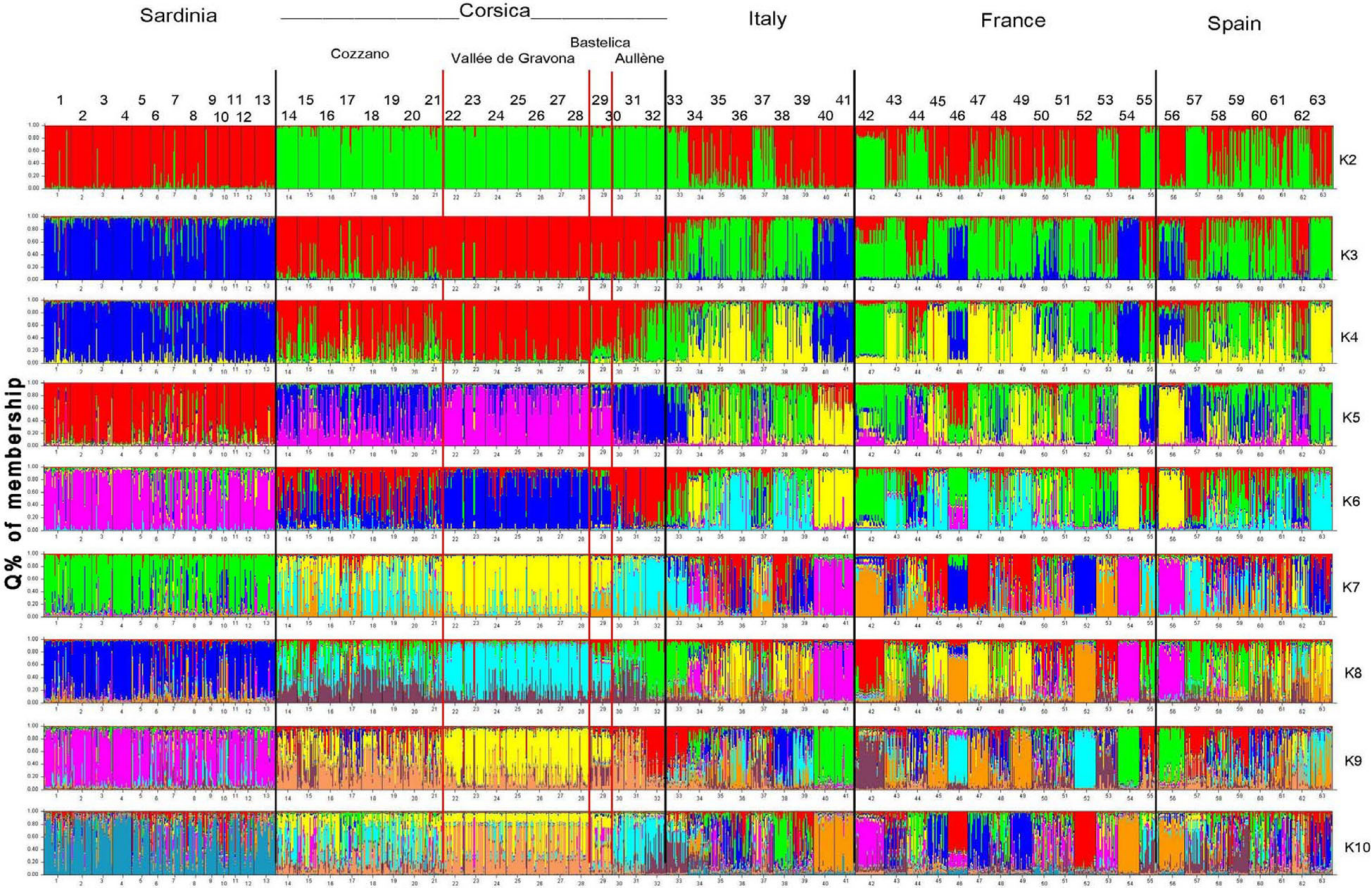
Assignment of 1424 larvae of *Trichinella britovi* on the basis of genotypic variation as determined by STRUCTURE. Parasites originated from the Mediterranean islands of Corsica (four localities) and Sardinia, and continental Italy, France and Spain. Larvae were analyzed independently, but are grouped for the purposes of display according to geographical origin. The analysis was performed assuming K = 2–10, assuming “admixture” as ancestry model, and “independent frequencies” as allele frequency model. Simulations were performed with a burn-in of 200,000 and 100,000 Markov Chain Monte Carlo iterations. Each individual larva is represented by its *Q* % of membership pattern (see Table 1 for isolate numbers)

The Bayesian analysis allowed us to assign each larva on the basis of their membership value (*Q*) to any K cluster superimposed by the algorithm, irrespective of the host from which they were isolated. This analysis was run assuming a range of K, and results were depicted for larvae organized by host origin (Fig. 3). The analysis of the post probability values [23] obtained by different K, did not show clear evidence of separate clusters. Nonetheless, the relationship of the intra-host genetic variability to region-wide genetic variability can be observed in the global analysis of K2-K10 simulations (Fig. 3).

From K = 2, Corsican and Sardinian isolates were separated into two clusters, whereas the continental isolates from Italy, France and Spain, showed a different genetic ancestry as displayed by color associations (Fig. 3). For example, for K = 5, isolate No. 46 from continental France was similar to those of Sardinia, recognizable by their blue color, and isolate Nos. 44 and 53 from continental France and No. 62 from continental Spain, recognizable by their magenta color, were genetically similar to those of Vallée de Gravona (Corsica) (Fig. 3). As the K value increased, the continental isolates showed high levels of intra-isolate complexity (genetic admixture) maintaining kinship relationships with isolates from other continental areas, e.g. isolate No. 40 and No. 41 from continental Italy with isolate No. 54 from continental France and isolate No. 56 from continental Spain, respectively.

**Fig. 4:**
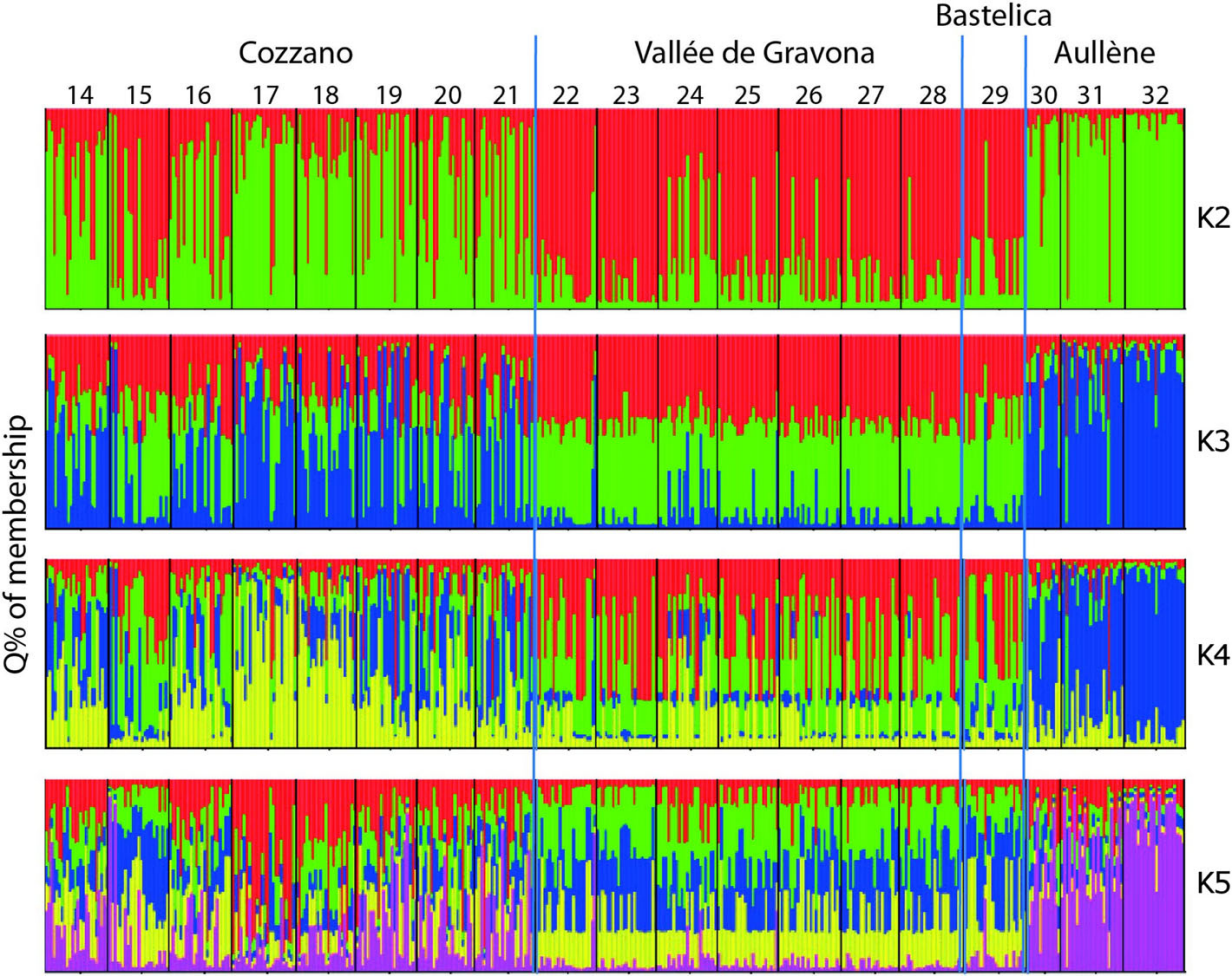
Bayesian analysis of *Trichinella britovi* isolates from Corsica (four localities). The numbers (14–32) are those of Table 1. Assignment of 432 larvae of *T. britovi* on the basis of genotypic variation was determined by STRUCTURE. Larvae were analyzed independently but are grouped for the purposes of display according to the locality of origin. The analysis was performed assuming K = 2–5, assuming “admixture” as ancestry model, and “independent frequencies” as allele frequency model. Simulations were performed with a burn-in of 200,000 and 100,000 Markov Chain Monte Carlo iterations. Each individual larva is represented by its *Q* % of membership pattern

The Bayesian analysis of the Corsican isolates (432 larvae) allowed us to understand the kinship affinity and net of interference with the other tested isolates (Fig. 4). Two separate clusters can be distinguished: (i) the isolates (Nos. 14–21) from Cozzano and those (Nos. 30–32) from Aullène; and (ii) the isolates (Nos. 22–28) from Vallée de Gravona and that (No. 29) from Bastelica (Fig. 4). The separation of the isolates of Cozzano from those of Vallée de Gravona was already evident for K = 2 (Cozzano with a green color pattern *versus* a red pattern of Vallée de Gravona), and further simulations with higher K values showed increasing admixture levels, but the separation did not change (Fig. 4).

The MLG of the isolate (No. 29) from Bastelica linked this isolate to those of Vallée de Gravona, independently of the K value, whereas the MLGs of the isolates (Nos. 31–32) of Aullène, while highlighting an admixture level lower than that of the Cozzano isolates, were linked with these isolates for K = 2 and K = 3 (Fig. 4). The MLG of the isolate No. 30 from “figatelli” marketed in Nice (mainland France), links this isolate to those of Cozzano (Fig. 4). In addition, isolate No. 30 from Nice showed a MLG pattern (for K = 2–5) very similar (low level of admixture) to that of isolate No. 31, which originated from the sausage collected from the breeder-butcher of Aullène, and to the MLG pattern of isolate No. 32, which originated from a domestic pig of Aullène (Fig. 4).

### Multidimensional analysis of *T. britovi* isolates

**Fig. 5:**
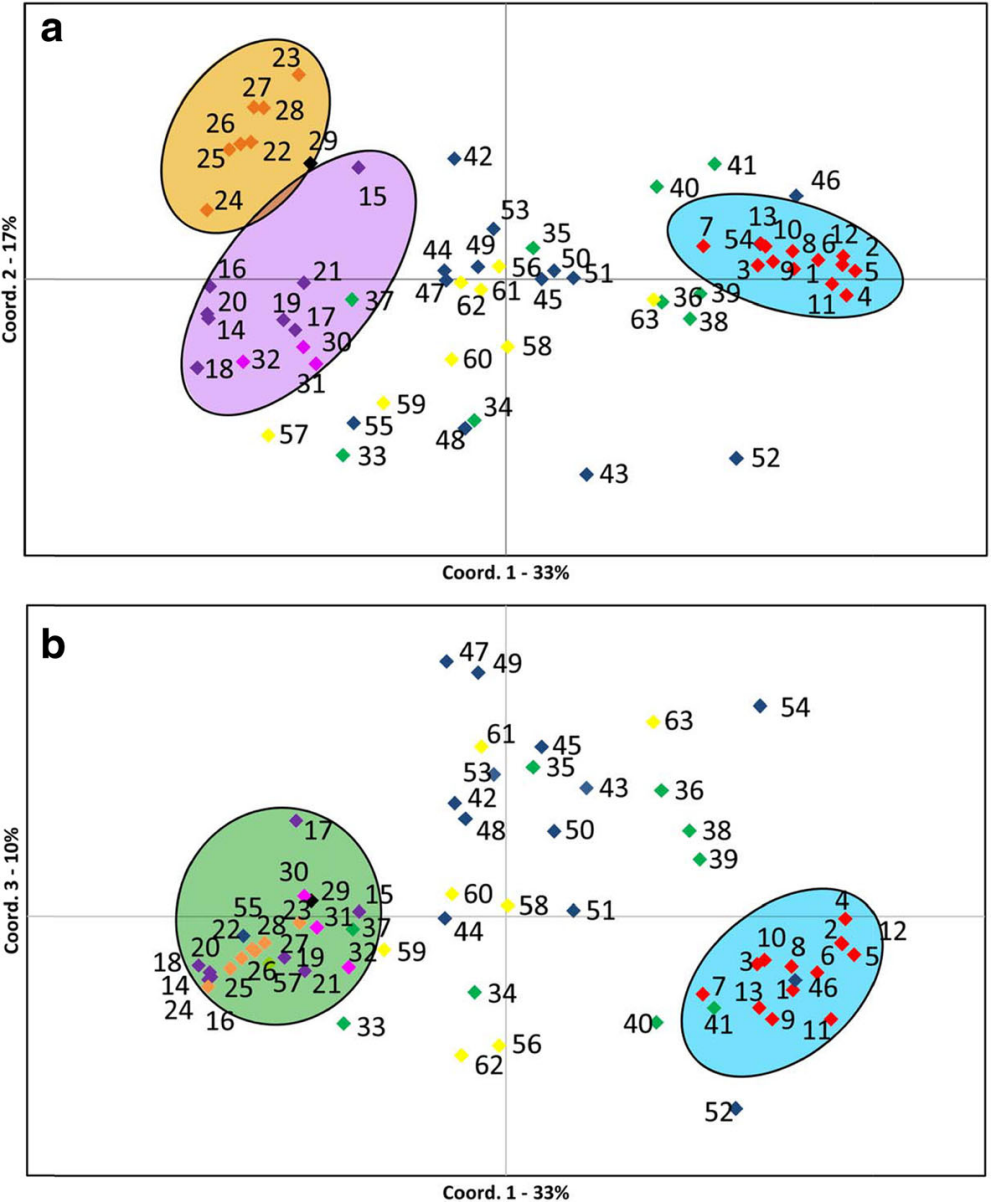
Principal component analysis of *Trichinella britovi* larval cohorts. Parasites derived from 63 animals (domestic pigs, wild boar, wolves, red foxes and meat products). **a** Coordinate 1 *vs* coordinate 2. Circles are drawn arbitrarily, but emphasize groupings of isolates of Corsica (Vallée de Gravona, yellow circle; Cozzano, violet circle) and Sardinia (blue circle), and of continental Europe (Italy, France and Spain). **b** Coordinate 1 *vs* coordinate 3. Circles are drawn arbitrarily, but emphasize groupings of isolates of Corsica (green circle) and Sardinia (blue circle), and of continental Europe (Italy, France and Spain) (see Table 1 for isolate numbers)

The PCoA of the 1424 MLGs identified three main axes, which represent about 60% of the total variance (Fig. 5). The first two axes (coordinate 1 = 33% and coordinate 2 = 17%; Fig. 5a) and the first and third axes (coordinate 1 = 33% and coordinate 3 = 10%; Fig. 5b) located the Sardinian isolates along the x-axis opposite to those from Corsica. Furthermore, the Corsican isolates were separated along the y-axis in the two clusters of Cozzano and Vallée de Gravona (Fig. 5a). It is noteworthy that the isolates from continental Italy, France and Spain, were distributed in the space between Corsican and Sardinian isolate clusters, without showing specific groupings and superimposing one another randomly (Fig. 5).

## Discussion

The differentiation of the MLG depicted by microsatellite analysis of *T. britovi* isolates originating from Corsica, Sardinia and continental Europe, does not support the assumption of a common recent origin for the parasite populations of the two islands as previously suspected [8], and rewrites the natural history of this zoonotic parasite in the two Mediterranean islands. Most likely, the almost simultaneous appearance of *T. britovi* in Corsica in 2004 and in Sardinia in 2005 was a mere coincidence.

Corsica was considered to be *Trichinella*-free due to the lack of reports of human or animal infections up until 2004 [7]. At the beginning of the 2000s, there was a heightened interest in monitoring *Trichinella* sp. infections in domestic pigs of the European Union with an improvement in detection methods involving a switch from trichinoscopy to the use of the more sensitive artificial digestion method and analyst training. From 2003 in France, including Corsica, a quality assurance programme was implemented for routine laboratories to monitor *Trichinella* testing in meat [26].

In Sardinia before the 2005 human outbreak, epidemiological investigations carried out using trichinoscopy, did not detect infection in either domestic or wild animals. Later, however, the analysis of the sample size and sampling localities of the surveys, suggested that methods used could have been inadequate to detect this parasite in the only focus discovered on the island so far [8].

The lack of epidemiological evidence on the circulation of *T. britovi* in Corsica and Sardinia before its appearance in 2004–2005 is not surprising since epidemiological investigations carried out by serology in Corsica, Italy and in the USA, showed that *Trichinella* spp. can circulate in a region with a larval burden in host muscles not detectable by direct methods (e.g. artificial digestion) and the presence of the parasite can be only inferred by indirect methods [7, 27–29]. In Ireland, the sylvatic cycle of *T. spiralis* existed for dozens of years independently of whether infection among domestic animals or humans had been demonstrated [30].

The wide variability of the genetic structure observed in the continental *T. britovi* isolates (Fig. 3) is probably related to the wide extension of the geographical area of sampling (Fig. 1) and to the limited host migration and genetic exchanges among isolates as observed in *T. spiralis* of the Extremadura region of Spain [12].

The *Q* membership values of *T. britovi* isolates of Corsica and Sardinia showed a substantial intra-isolate MLG homogeneity and they appear differentiated from one another irrespective of the K value (Fig. 3) and from continental *T. britovi* isolates, where a higher level of admixture occurs. However, with increasing K values, several larvae from the two islands showed admixture with larvae of continental areas (e.g. K = 5, Sardinia *vs* France No. 46; Corsica *vs* France No. 44 and Spain No. 57) (Fig. 3).

The multidimensional analysis supports that Corsica and Sardinia harbor genetically differentiate isolates over all the three main axes (Fig. 5), while the continental isolates are overdispersed in the three-dimensional space (Fig. 5), as it is likely that the large distances among sampled continental isolates are a factor limiting the gene flow.

Both Bayesian and PCoA independent algorithms show two *T. britovi* clusters segregated in remote valleys of Corsica (Figs. 4 and 5a), suggesting a low genetic exchange among them due to the island orography and likely human behavior.

According to Bayesian and PCoA analyses, the Corsican isolates are separated into two clusters related to Cozzano and Vallée de Gravona. On the basis of presence/absence of alleles, the seven isolates of Vallée de Gravona are fixed to one of the four alleles identified in the Cozzano valley (TS1010B/240; TS1380/297) (Fig. 2, Additional file 2: Table S2). Alleles detected in the Bastelica isolate are fixed for the same alleles (TS1010B/240, TS1380/297) as observed in Vallée de Gravona (Fig. 2, Additional file 2: Table S2). Alleles detected in the three Aullène isolates resemble those detected in Cozzano (Fig. 2, Additional file 2: Table S2). The genetic structure observed by the Bayesian analysis confirms the associations between Bastelica and Vallée de Gravona isolates, as well as between Aullène and Cozzano isolates, since they are assigned to the same clusters for all K values (Fig. 4). The homogeneity of the *Q* membership of individuals suggests that genetic drift affected the allele frequencies of the loci of the Aullène isolates. The MLG analysis of the 31 continental *T. britovi* isolates appears to be inadequate to identify the European region of origin of the *T. britovi* populations detected in the two Mediterranean islands. The sharing of one or more alleles between two geographical populations only, could suggest that one population derives from the other. Based on the presence/absence of alleles in *T. britovi* populations of Corsica and Sardinia, we observed that Corsica shares one allele (TS1380/291) with continental Spain, whereas Sardinia shares two alleles (TS1380/267, TB1019/292) with continental France and one allele (TB1019/289) with continental Italy (Additional file 2: Table S2). We can speculate that *T. britovi* populations of the two islands did not derive from each other at least in recent times. Furthermore, the allele sharing suggests that the Corsica population could originate from Spain and the Sardinia population could originate from France/Italy. The two island populations could have separately evolved as unique genetic structures different to those typical of *T. britovi* continental populations.

The MLG analysis of *T. britovi* larvae isolated from figatelli (isolate No. 30) marketed in the Nice region (the South of France), where they caused a human outbreak, links their origin with larvae isolated from a sausage (isolate No. 31) collected at the breeder-butcher of Aullène and with larvae from a domestic pig (isolate No. 32) reared in Aullène (Corsica) (Fig. 4). This result supports the epidemiological investigation carried out following the outbreak [9]. In addition, the MLG analysis suggests that larvae isolated from pork delicatessen produced in Aullène and those from the domestic pig of Aullène originated from the Cozzano focus. It follows that the MLG analysis could be a powerful tool to trace “from fork to farm” the origin of *T. britovi* infections and the trade of infected animals and/or food.

The red fox is one of the most important natural reservoir hosts of *T. britovi* in Europe [31]. Today, the Corsican and Sardinian foxes are recognized as a subspecies (*Vulpes vulpes ichnusae*) of the continental population. This canid colonized these Mediterranean islands during the Middle Pleistocene and Early Holocene (from 700 to 11,000 B.C.) when a terrestrial bridge allowed the migration of mammals from continental Italy to the two islands [32]. Since *T. britovi* probably diversified from an ancestral species in the Pleistocene [33, 34], we can speculate that foxes, which colonized the two islands, were infected by this parasite. In Corsica and Sardinia, hunters do not collect fox carcasses after hunting, thus favoring the transmission of *T. britovi* to wild boar and domestic pigs as extensive breeding is a common practice on both islands.

Other carnivores present in Corsica and Sardinia such as the wild cat (*Felis lybica sarda*), probably introduced by Phoenicians between the 9th and 3rd century B.C. [35], the marten (*Martes martes*) and the weasel (*Mustela nivalis*), do not play an important role as reservoir hosts, and *T. britovi* has been not detected in these species of the two islands so far.

The wild boar of Corsica and Sardinia has been described as a subspecies (*Sus scrofa meridionalis*), which was not present on these islands before the 7000 B.C. and may have originated from domestic pigs introduced by humans, an origin from independently domesticated swine on the Italian mainland that have since gone feral [36]. Even though domestic and wild pigs cannot be considered as good reservoir hosts for *T. britovi* [1, 31], they could have introduced *T. britovi* in Corsica and Sardinia in ancient times.

At the end of the 1970s, the near disappearance of the wild boar population in Corsica due to classical swine fever was followed by the introduction of wild boar from the Ardennes, mainland France [37]. Today, Corsica has the highest density of wild boar and free ranging pig populations of France [38].

Finally, hunting dogs can also be considered as a possible source of infection for domestic and wild swine, since *T. britovi* larvae and/or anti-*Trichinella* antibodies have been detected in these animals [29]. In Corsica, epidemiological evidence suggests that when a hunting dog dies during hunting, its carcass is usually used to feed domestic pigs (Vallée I., unpublished data).

## Conclusions

This microsatellite analysis suggests that *T. britovi* was introduced to the two Mediterranean islands of Sardinia and Corsica by two or more independent events and that gene flow among the *T. britovi* isolates of Corsica is very limited and restricted to small foci present in the deep valleys. Furthermore, the MLG analysis proved to be a useful tool to trace “from fork to farm” the *T. britovi* infection source. The MLG analysis may be a suitable method to describe the natural history of insular populations of *T. britovi*, supporting epidemiological investigations during outbreaks, whereas its use at the continental level could be more difficult due to the limited amount of data. Therefore, the screening of a larger number of *T. britovi* isolates using the MLG analysis will be necessary to provide a useful tool for epidemiological investigations within continental areas.

## Additional files

Additional file 1: **Table S1.** Genetic variability of 63 *Trichinella britovi* isolates from two Mediterranean islands and three continental regions. (PDF 89 kb)

Additional file 2: **Table S2.** Allele frequencies and sample size of *Trichinella britovi* larval cohorts derived from 63 animals. (PDF 53 kb)

Additional file 3: **Table S3.** Pairwise Fst values of the 63 *Trichinella britovi* isolates. (PDF 240 kb)

## Abbreviations

%Pl: Proportion of polymorphic loci
He: Unbiased expected heterozygosity
Ho: Observed heterozygosity
HWE: Hardy-Weinberg expectations
MLG: Multilocus genotype
Na: Mean number of alleles per locus
Ne: Effective number of alleles
PCoA: Principal coordinates analysis
*Q*: Estimated membership coefficients
