# Supplementary material for: Multilocus genotype analysis outlines distinct histories for Trichinella britovi in the neighboring Mediterranean islands of Corsica and Sardinia
Source: Parasit Vectors. 2018 Jun 19;11:353. doi: 10.1186/s13071-018-2939-9 (PMC6006749; doi:10.1186/s13071-018-2939-9)
Supplement: Supplementary file 1 — Table S1. Genetic variability of 63 Trichinella britovi isolates from two Mediterranean islands and three continental regions. (PDF 89 kb) [file 13071_2018_2939_MOESM1_ESM.pdf]

Additional file 1: Table S1

Genetic variability of 63 *Trichinella britovi* isolates from different hosts of the Sardinia island (No. 1-13), Corsica island (No. 14-32), continental Italy (No. 33-41), continental France (No. 42-55), and continental Spain (No. 56-63). Average values for four microsatellite loci for each of the 63 isolates: N = individual larvae studied; Na = number of different alleles; Ne = number of effective alleles; Ho = observed heterozygosity; He = expected heterozygosity. Fis = inbreeding coefficient; %Pl = proportion of polymorphic loci.

No.	Mean (SE)					%Pl	Fis
	N	Na	Ne	Ho	He		
1	25.250 (3.425)	3.250 (0.250)	2.312 (0.260)	0.567 (0.063)	0.552 (0.047)	100	-0.005
2	23.000 (0.707)	2.750 (0.479)	2.222 (0.223)	0.515 (0.079)	0.535 (0.049)	100	0.060
3	22.000 (0.707)	4.000 (0.408)	2.753 (0.524)	0.571 (0.066)	0.602 (0.061)	100	0.076
4	20.750 (0.250)	2.500 (0.289)	2.287 (0.233)	0.636 (0.074)	0.550 (0.042)	100	-0.132
5	19.750 (0.250)	3.250 (0.629)	2.042 (0.184)	0.505 (0.051)	0.497 (0.050)	100	-0.041
6	14.000 (0.000)	2.750 (0.250)	1.859 (0.116)	0.500 (0.065)	0.455 (0.036)	100	-0.153
7	24.000 (0.000)	3.750 (0.250)	2.391 (0.315)	0.573 (0.046)	0.558 (0.063)	100	-0.056
8	21.750 (0.250)	3.250 (0.250)	2.196 (0.141)	0.542 (0.053)	0.540 (0.027)	100	0.050
9	13.750 (0.250)	3.250 (0.250)	2.243 (0.270)	0.492 (0.047)	0.535 (0.052)	100	0.073
10	13.000 (0.000)	3.750 (0.479)	2.387 (0.490)	0.558 (0.073)	0.538 (0.070)	100	0.007
11	13.000 (0.000)	3.250 (0.629)	2.281 (0.577)	0.423 (0.080)	0.493 (0.093)	100	0.158
12	15.000 (0.000)	3.250 (0.250)	2.147 (0.238)	0.417 (0.074)	0.513 (0.063)	100	0.221[a]
13	23.750 (0.250)	3.500 (0.289)	2.371 (0.414)	0.559 (0.058)	0.544 (0.067)	100	-0.006
14	22.250 (1.031)	2.500 (0.289)	1.571 (0.321)	0.294 (0.144)	0.293 (0.117)	100	0.021
15	22.750 (0.250)	2.000 (0.408)	1.594 (0.356)	0.246 (0.124)	0.290 (0.128)	75	0.176
16	22.500 (0.957)	2.250 (0.479)	1.537 (0.276)	0.242 (0.127)	0.282 (0.127)	75	0.165
17	23.000 (0.707)	2.250 (0.479)	1.822 (0.322)	0.309 (0.106)	0.382 (0.134)	75	0.215[a]
18	21.250 (1.181)	1.750 (0.250)	1.319 (0.140)	0.165 (0.095)	0.215 (0.086)	75	0.258[a]
19	21.000 (2.000)	2.750 (0.250)	1.700 (0.141)	0.338 (0.036)	0.401 (0.044)	100	0.182[a]
20	21.250 (0.479)	2.250 (0.479)	1.556 (0.311)	0.290 (0.137)	0.284 (0.126)	75	0.004
21	21.750 (0.479)	2.750 (0.479)	1.773 (0.310)	0.365 (0.122)	0.383 (0.104)	100	0.070
22	22.500 (0.500)	1.750 (0.479)	1.443 (0.263)	0.166 (0.123)	0.233 (0.136)	50	0.309[a]
23	23.000 (0.000)	1.750 (0.479)	1.243 (0.186)	0.120 (0.074)	0.150 (0.104)	50	0.222
24	22.500 (0.500)	1.500 (0.289)	1.313 (0.233)	0.179 (0.114)	0.176 (0.117)	50	0.008
25	22.500 (0.500)	1.500 (0.289)	1.299 (0.206)	0.167 (0.113)	0.178 (0.112)	50	0.082
26	23.500 (0.500)	1.500 (0.289)	1.392 (0.227)	0.217 (0.126)	0.220 (0.127)	50	0.036
27	21.750 (0.250)	1.500 (0.289)	1.262 (0.152)	0.174 (0.101)	0.172 (0.099)	50	0.011
28	23.750 (0.250)	1.750 (0.479)	1.324 (0.198)	0.170 (0.098)	0.194 (0.114)	50	0.144
29	23.500 (0.500)	1.750 (0.479)	1.622 (0.445)	0.229 (0.133)	0.257 (0.159)	50	0.131
30	11.250 (1.436)	2.500 (0.289)	1.878 (0.311)	0.381 (0.046)	0.429 (0.078)	100	0.156
31	23.500 (0.289)	2.750 (0.250)	1.882 (0.299)	0.395 (0.117)	0.434 (0.075)	100	0.110
32	22.750 (0.250)	1.750 (0.250)	1.459 (0.214)	0.321 (0.140)	0.265 (0.112)	75	-0.189
33	23.000 (1.000)	2.000 (0.408)	1.433 (0.258)	0.252 (0.137)	0.234 (0.127)	75	-0.052
34	21.500 (0.866)	2.250 (0.629)	1.536 (0.192)	0.308 (0.105)	0.309 (0.106)	75	0.028
35	22.000 (0.577)	3.250 (0.479)	2.283 (0.365)	0.512 (0.074)	0.528 (0.072)	100	0.053
36	23.500 (0.289)	2.750 (0.479)	2.328 (0.224)	0.520 (0.031)	0.558 (0.042)	100	0.090
37	22.500 (1.190)	3.750 (0.854)	2.445 (0.833)	0.473 (0.115)	0.460 (0.135)	100	-0.004
38	21.250 (0.479)	3.500 (0.289)	2.943 (0.224)	0.501 (0.081)	0.654 (0.028)	100	0.257[a]
39	21.500 (1.500)	4.250 (1.031)	2.529 (0.363)	0.636 (0.050)	0.581 (0.056)	100	-0.070
40	22.750 (0.250)	2.000 (0.000)	1.429 (0.115)	0.252 (0.065)	0.284 (0.068)	100	0.134
41	22.750 (0.250)	2.250 (0.250)	1.316 (0.109)	0.230 (0.069)	0.226 (0.057)	100	0.002
42	30.750 (1.250)	1.500 (0.289)	1.219 (0.170)	0.154 (0.111)	0.139 (0.098)	50	-0.096
43	24.000 (0.000)	2.000 (0.000)	1.537 (0.260)	0.271 (0.132)	0.288 (0.122)	100	0.080
44	22.000 (1.000)	2.500 (0.289)	2.181 (0.274)	0.501 (0.045)	0.522 (0.053)	100	0.063
45	22.000 (0.408)	3.500 (0.289)	2.140 (0.181)	0.525 (0.051)	0.523 (0.040)	100	0.018
46	20.750 (0.946)	1.750 (0.250)	1.081 (0.038)	0.076 (0.035)	0.072 (0.032)	75	-0.041
47	22.250 (0.750)	2.750 (0.479)	1.959 (0.297)	0.294 (0.076)	0.441 (0.109)	100	0.354[a]
48	21.750 (1.652)	3.250 (0.479)	1.963 (0.342)	0.392 (0.090)	0.438 (0.105)	100	0.128
49	23.750 (0.250)	2.500 (0.500)	1.639 (0.235)	0.308 (0.099)	0.344 (0.109)	100	0.127
50	24.000 (0.000)	4.500 (1.190)	2.736 (0.302)	0.646 (0.055)	0.621 (0.042)	100	-0.019
51	22.250 (0.750)	3.750 (0.479)	2.438 (0.356)	0.565 (0.064)	0.562 (0.065)	100	0.019
52	23.500 (0.500)	1.500 (0.289)	1.241 (0.147)	0.117 (0.079)	0.161 (0.095)	50	0.290[a]
53	23.750 (0.250)	2.250 (0.250)	1.918 (0.294)	0.444 (0.068)	0.445 (0.073)	100	0.025
54	23.750 (0.250)	2.500 (0.645)	1.711 (0.390)	0.361 (0.142)	0.330 (0.132)	75	-0.073
55	21.000 (0.000)	4.000 (1.080)	1.881 (0.599)	0.345 (0.150)	0.338 (0.142)	100	0.003
56	26.000 (3.000)	3.000 (0.408)	1.574 (0.268)	0.383 (0.165)	0.303 (0.121)	100	-0.245[a]
57	23.750 (0.250)	1.750 (0.479)	1.423 (0.336)	0.182 (0.115)	0.201 (0.138)	50	0.116
58	23.500 (0.500)	2.750 (0.479)	2.157 (0.294)	0.436 (0.110)	0.509 (0.067)	100	0.165[a]
59	20.000 (3.000)	2.500 (0.289)	1.586 (0.266)	0.274 (0.071)	0.321 (0.098)	100	0.172
60	23.000 (0.000)	3.500 (0.645)	2.366 (0.359)	0.533 (0.099)	0.552 (0.056)	100	0.057
61	22.750 (0.946)	3.250 (0.250)	2.002 (0.274)	0.439 (0.054)	0.471 (0.071)	100	0.090
62	19.000 (2.000)	2.500 (0.645)	1.958 (0.402)	0.418 (0.160)	0.407 (0.142)	75	0.004
63	22.250 (1.750)	2.750 (0.479)	2.239 (0.202)	0.528 (0.052)	0.542 (0.041)	100	0.049
Total	21.774 (0.230)	2.655 (0.071)	1.868 (0.044)	0.373 (0.014)	0.389 (0.014)	0.837(0.02)	0.038 (0.013)

[a] Significant deviation from Hardy-Weinberg expectation (P < to corrected nominal level).
