# Supplementary material for: Multilocus genotype analysis outlines distinct histories for Trichinella britovi in the neighboring Mediterranean islands of Corsica and Sardinia
Source: Parasit Vectors. 2018 Jun 19;11:353. doi: 10.1186/s13071-018-2939-9 (PMC6006749; doi:10.1186/s13071-018-2939-9)
Supplement: Supplementary file 2 — Table S2. Allele frequencies and sample size of Trichinella britovi larval cohorts derived from 63 animals. (PDF 53 kb) [file 13071_2018_2939_MOESM2_ESM.pdf]

Additional file 2: Table S2

Allele frequencies and sample size by populations of *Trichinella britovi* larval cohorts derived from 63 animals of Corsica (four localities) and Sardinia islands, and of continental Italy, France and Spain. For the isolate numbers see Table 1.

			SARDINIA													CORSICA – Cozzano							
Locus	Isolate code		1	2	3	4	5	6	7	8	9	10	11	12	13	14	15	16	17	18	19	20	21
TS1010B	Sample size		28	24	23	21	20	14	24	22	14	13	13	15	24	24	23	24	24	23	23	22	23
	Allele	230	0.000	0.000	0.087	0.000	0.000	0.000	0.000	0.000	0.000	0.000	0.000	0.000	0.000	0.000	0.130	0.000	0.000	0.000	0.152	0.000	0.043
		238	0.714	0.708	0.391	0.595	0.775	0.786	0.583	0.636	0.714	0.731	0.808	0.800	0.563	0.042	0.000	0.000	0.000	0.000	0.043	0.000	0.087
		240	0.089	0.000	0.065	0.000	0.025	0.143	0.250	0.068	0.143	0.154	0.038	0.033	0.042	0.958	0.870	1.000	1.000	1.000	0.804	1.000	0.826
		242	0.196	0.292	0.457	0.405	0.200	0.071	0.167	0.295	0.143	0.115	0.154	0.167	0.396	0.000	0.000	0.000	0.000	0.000	0.000	0.000	0.043
		244	0.000	0.000	0.000	0.000	0.000	0.000	0.000	0.000	0.000	0.000	0.000	0.000	0.000	0.000	0.000	0.000	0.000	0.000	0.000	0.000	0.000
		246	0.000	0.000	0.000	0.000	0.000	0.000	0.000	0.000	0.000	0.000	0.000	0.000	0.000	0.000	0.000	0.000	0.000	0.000	0.000	0.000	0.000
		248	0.000	0.000	0.000	0.000	0.000	0.000	0.000	0.000	0.000	0.000	0.000	0.000	0.000	0.000	0.000	0.000	0.000	0.000	0.000	0.000	0.000
TS1380	Sample size		29	24	23	21	20	14	24	22	14	13	13	15	24	20	23	20	24	18	23	21	21
	Allele	267	0.000	0.000	0.087	0.000	0.000	0.000	0.000	0.000	0.000	0.000	0.000	0.000	0.000	0.000	0.000	0.000	0.000	0.000	0.000	0.000	0.000
		276	0.000	0.000	0.000	0.000	0.000	0.000	0.000	0.000	0.000	0.000	0.000	0.000	0.000	0.000	0.000	0.000	0.000	0.000	0.000	0.000	0.000
		282	0.224	0.208	0.022	0.310	0.150	0.107	0.125	0.205	0.143	0.077	0.231	0.333	0.208	0.025	0.000	0.050	0.438	0.194	0.130	0.071	0.071
		285	0.138	0.271	0.261	0.286	0.275	0.179	0.083	0.136	0.143	0.231	0.115	0.133	0.042	0.000	0.000	0.025	0.125	0.000	0.000	0.000	0.000
		291	0.000	0.000	0.000	0.000	0.000	0.000	0.000	0.000	0.000	0.000	0.000	0.000	0.000	0.050	0.000	0.000	0.000	0.000	0.087	0.024	0.000
		297	0.638	0.521	0.630	0.405	0.575	0.714	0.771	0.659	0.714	0.692	0.654	0.533	0.750	0.925	1.000	0.925	0.438	0.806	0.783	0.905	0.929
		300	0.000	0.000	0.000	0.000	0.000	0.000	0.021	0.000	0.000	0.000	0.000	0.000	0.000	0.000	0.000	0.000	0.000	0.000	0.000	0.000	0.000
		312	0.000	0.000	0.000	0.000	0.000	0.000	0.000	0.000	0.000	0.000	0.000	0.000	0.000	0.000	0.000	0.000	0.000	0.000	0.000	0.000	0.000
TB1019	Sample size		15	23	22	21	20	14	24	22	14	13	13	15	24	24	23	24	23	23	23	22	21
	Allele	272	0.000	0.000	0.000	0.000	0.025	0.036	0.083	0.045	0.000	0.077	0.000	0.000	0.021	0.771	0.196	0.667	0.717	0.913	0.739	0.750	0.595
		277	0.533	0.522	0.636	0.571	0.450	0.393	0.354	0.409	0.500	0.192	0.731	0.333	0.458	0.000	0.000	0.000	0.000	0.000	0.000	0.000	0.000
		283	0.367	0.478	0.318	0.143	0.475	0.571	0.521	0.545	0.393	0.654	0.269	0.600	0.500	0.229	0.804	0.333	0.283	0.087	0.261	0.250	0.405
		286	0.000	0.000	0.000	0.000	0.025	0.000	0.000	0.000	0.000	0.000	0.000	0.000	0.021	0.000	0.000	0.000	0.000	0.000	0.000	0.000	0.000
		289	0.100	0.000	0.000	0.000	0.000	0.000	0.000	0.000	0.000	0.000	0.000	0.000	0.000	0.000	0.000	0.000	0.000	0.000	0.000	0.000	0.000
		292	0.000	0.000	0.045	0.286	0.025	0.000	0.042	0.000	0.107	0.077	0.000	0.067	0.000	0.000	0.000	0.000	0.000	0.000	0.000	0.000	0.000
TB922	Sample size		29	21	20	20	19	14	24	21	13	13	13	15	23	21	22	22	21	21	15	20	22
	Allele	237	0.000	0.000	0.000	0.000	0.000	0.000	0.000	0.000	0.000	0.000	0.000	0.000	0.000	0.000	0.000	0.000	0.000	0.000	0.000	0.000	0.000
		239	0.431	0.452	0.325	0.575	0.579	0.500	0.396	0.452	0.500	0.385	0.385	0.467	0.370	0.000	0.000	0.000	0.000	0.000	0.000	0.000	0.000
		241	0.034	0.000	0.125	0.000	0.000	0.000	0.042	0.000	0.000	0.115	0.115	0.000	0.000	0.000	0.000	0.000	0.000	0.000	0.000	0.000	0.000
		243	0.000	0.024	0.125	0.000	0.000	0.000	0.000	0.024	0.154	0.115	0.115	0.033	0.130	0.000	0.000	0.000	0.000	0.000	0.000	0.000	0.000
		245	0.000	0.000	0.000	0.000	0.000	0.000	0.000	0.000	0.000	0.000	0.000	0.000	0.000	0.452	0.455	0.477	0.167	0.262	0.433	0.450	0.523
		247	0.241	0.119	0.150	0.000	0.000	0.000	0.271	0.119	0.231	0.115	0.154	0.100	0.217	0.429	0.159	0.477	0.643	0.738	0.533	0.450	0.273
		249	0.293	0.405	0.275	0.425	0.421	0.500	0.292	0.405	0.115	0.269	0.231	0.400	0.283	0.119	0.386	0.045	0.190	0.000	0.033	0.100	0.205
		251	0.000	0.000	0.000	0.000	0.000	0.000	0.000	0.000	0.000	0.000	0.000	0.000	0.000	0.000	0.000	0.000	0.000	0.000	0.000	0.000	0.000
		253	0.000	0.000	0.000	0.000	0.000	0.000	0.000	0.000	0.000	0.000	0.000	0.000	0.000	0.000	0.000	0.000	0.000	0.000	0.000	0.000	0.000

			CORSICA – Vallée de Gravona							Bastelica	Aullène			ITALY								
Locus	Isolate code		22	23	24	25	26	27	28	29	30	31	32	33	34	35	36	37	38	39	40	41
TS1010B	Sample size		23	23	23	23	24	22	24	24	12	24	23	24	23	23	23	24	21	23	23	22
	Allele	230	0.000	0.000	0.000	0.000	0.000	0.000	0.000	0.000	0.125	0.229	0.326	0.000	0.000	0.000	0.000	0.000	0.000	0.000	0.000	0.000
		238	0.000	0.000	0.000	0.000	0.000	0.000	0.000	0.000	0.042	0.021	0.000	0.250	0.348	0.174	0.565	0.125	0.476	0.674	0.000	0.023
		240	1.000	1.000	1.000	1.000	1.000	1.000	1.000	1.000	0.833	0.750	0.674	0.750	0.652	0.761	0.435	0.854	0.095	0.196	0.239	0.068
		242	0.000	0.000	0.000	0.000	0.000	0.000	0.000	0.000	0.000	0.000	0.000	0.000	0.000	0.065	0.000	0.021	0.429	0.065	0.761	0.909
		244	0.000	0.000	0.000	0.000	0.000	0.000	0.000	0.000	0.000	0.000	0.000	0.000	0.000	0.000	0.000	0.000	0.000	0.065	0.000	0.000
		246	0.000	0.000	0.000	0.000	0.000	0.000	0.000	0.000	0.000	0.000	0.000	0.000	0.000	0.000	0.000	0.000	0.000	0.000	0.000	0.000
		248	0.000	0.000	0.000	0.000	0.000	0.000	0.000	0.000	0.000	0.000	0.000	0.000	0.000	0.000	0.000	0.000	0.000	0.000	0.000	0.000
TS1380	Sample size		23	23	23	23	24	22	24	24	13	23	23	24	23	23	24	23	22	23	23	23
	Allele	267	0.000	0.000	0.000	0.000	0.000	0.000	0.000	0.000	0.000	0.000	0.000	0.000	0.000	0.000	0.000	0.000	0.000	0.000	0.000	0.000
		276	0.000	0.000	0.000	0.000	0.000	0.000	0.000	0.000	0.000	0.000	0.000	0.000	0.000	0.000	0.000	0.000	0.000	0.000	0.000	0.000
		282	0.000	0.000	0.000	0.000	0.000	0.000	0.000	0.000	0.000	0.022	0.000	0.000	0.217	0.304	0.083	0.130	0.318	0.130	0.043	0.065
		285	0.000	0.000	0.000	0.000	0.000	0.000	0.000	0.000	0.000	0.000	0.000	0.000	0.000	0.065	0.354	0.000	0.182	0.043	0.000	0.000
		291	0.000	0.000	0.000	0.000	0.000	0.000	0.000	0.000	0.346	0.283	0.087	0.000	0.000	0.000	0.000	0.000	0.000	0.000	0.000	0.000
		297	1.000	1.000	1.000	1.000	1.000	1.000	1.000	1.000	0.654	0.696	0.913	1.000	0.783	0.413	0.083	0.870	0.114	0.457	0.957	0.935
		300	0.000	0.000	0.000	0.000	0.000	0.000	0.000	0.000	0.000	0.000	0.000	0.000	0.000	0.217	0.479	0.000	0.386	0.370	0.000	0.000
		312	0.000	0.000	0.000	0.000	0.000	0.000	0.000	0.000	0.000	0.000	0.000	0.000	0.000	0.000	0.000	0.000	0.000	0.000	0.000	0.000
TB1019	Sample size		23	23	23	23	24	22	24	24	7	24	23	24	20	21	24	24	20	23	23	23
	Allele	272	0.304	0.087	0.543	0.370	0.313	0.227	0.208	0.250	0.786	0.833	1.000	0.979	1.000	0.310	0.396	0.563	0.325	0.391	0.239	0.130
		277	0.000	0.000	0.000	0.000	0.000	0.000	0.000	0.000	0.000	0.000	0.000	0.000	0.000	0.000	0.000	0.000	0.000	0.000	0.000	0.000
		283	0.696	0.913	0.457	0.630	0.688	0.773	0.792	0.750	0.214	0.167	0.000	0.000	0.000	0.690	0.604	0.333	0.225	0.609	0.761	0.870
		286	0.000	0.000	0.000	0.000	0.000	0.000	0.000	0.000	0.000	0.000	0.000	0.000	0.000	0.000	0.000	0.083	0.450	0.000	0.000	0.000
		289	0.000	0.000	0.000	0.000	0.000	0.000	0.000	0.000	0.000	0.000	0.000	0.021	0.000	0.000	0.000	0.021	0.000	0.000	0.000	0.000
		292	0.000	0.000	0.000	0.000	0.000	0.000	0.000	0.000	0.000	0.000	0.000	0.000	0.000	0.000	0.000	0.000	0.000	0.000	0.000	0.000
TB922	Sample size		21	23	21	21	22	21	23	22	13	23	22	20	20	21	23	19	22	17	22	23
	Allele	237	0.000	0.000	0.000	0.000	0.000	0.000	0.000	0.000	0.000	0.000	0.000	0.000	0.000	0.000	0.000	0.000	0.000	0.000	0.000	0.000
		239	0.000	0.000	0.000	0.000	0.000	0.000	0.000	0.000	0.000	0.000	0.000	0.000	0.725	0.262	0.348	0.000	0.000	0.088	0.795	0.739
		241	0.000	0.000	0.000	0.000	0.000	0.000	0.000	0.000	0.000	0.000	0.000	0.450	0.050	0.071	0.457	0.000	0.273	0.265	0.000	0.000
		243	0.000	0.000	0.000	0.000	0.000	0.000	0.000	0.000	0.000	0.000	0.000	0.000	0.150	0.000	0.000	0.079	0.000	0.000	0.000	0.000
		245	0.262	0.196	0.119	0.143	0.341	0.214	0.261	0.409	0.308	0.370	0.364	0.525	0.000	0.000	0.196	0.211	0.000	0.029	0.000	0.000
		247	0.643	0.717	0.881	0.857	0.659	0.786	0.696	0.250	0.462	0.435	0.000	0.000	0.000	0.000	0.000	0.079	0.364	0.029	0.000	0.000
		249	0.095	0.087	0.000	0.000	0.000	0.000	0.043	0.341	0.231	0.196	0.636	0.025	0.075	0.548	0.000	0.263	0.318	0.441	0.205	0.261
		251	0.000	0.000	0.000	0.000	0.000	0.000	0.000	0.000	0.000	0.000	0.000	0.000	0.000	0.119	0.000	0.105	0.045	0.029	0.000	0.000
		253	0.000	0.000	0.000	0.000	0.000	0.000	0.000	0.000	0.000	0.000	0.000	0.000	0.000	0.000	0.000	0.263	0.000	0.118	0.000	0.000

			FRANCE														SPAIN							
Locus	Isolate code		42	43	44	45	46	47	48	49	50	51	52	53	54	55	56	57	58	59	60	61	62	63
TS1010B	Sample size		32	24	23	23	22	23	24	24	24	23	24	24	24	21	29	24	24	23	23	24	21	24
	Allele	230	0.000	0.000	0.000	0.000	0.000	0.000	0.000	0.000	0.000	0.043	0.000	0.000	0.000	0.024	0.000	0.000	0.000	0.000	0.000	0.000	0.000	0.000
		238	0.000	0.542	0.326	0.239	1.000	0.065	0.188	0.021	0.125	0.348	1.000	0.208	0.083	0.000	0.017	0.000	0.146	0.261	0.370	0.104	0.357	0.438
		240	1.000	0.458	0.674	0.717	0.000	0.935	0.646	0.979	0.646	0.500	0.000	0.792	0.104	0.976	0.586	1.000	0.438	0.739	0.630	0.813	0.286	0.417
		242	0.000	0.000	0.000	0.043	0.000	0.000	0.042	0.000	0.229	0.043	0.000	0.000	0.813	0.000	0.397	0.000	0.417	0.000	0.000	0.000	0.000	0.000
		244	0.000	0.000	0.000	0.000	0.000	0.000	0.000	0.000	0.000	0.065	0.000	0.000	0.000	0.000	0.000	0.000	0.000	0.000	0.000	0.083	0.000	0.146
		246	0.000	0.000	0.000	0.000	0.000	0.000	0.125	0.000	0.000	0.000	0.000	0.000	0.000	0.000	0.000	0.000	0.000	0.000	0.000	0.000	0.000	0.000
		248	0.000	0.000	0.000	0.000	0.000	0.000	0.000	0.000	0.000	0.000	0.000	0.000	0.000	0.000	0.000	0.000	0.000	0.000	0.000	0.000	0.357	0.000
TS1380	Sample size		32	24	23	22	21	23	22	24	24	23	24	24	24	21	29	24	22	11	23	23	21	24
	Allele	267	0.000	0.000	0.000	0.000	0.000	0.000	0.023	0.000	0.000	0.000	0.000	0.000	0.000	0.000	0.000	0.000	0.000	0.000	0.000	0.000	0.000	0.000
		276	0.000	0.000	0.000	0.000	0.000	0.000	0.000	0.000	0.000	0.000	0.000	0.000	0.000	0.000	0.000	0.000	0.000	0.045	0.000	0.000	0.000	0.000
		282	0.000	0.000	0.326	0.136	0.000	0.043	0.182	0.000	0.271	0.239	0.000	0.583	0.000	0.119	0.000	0.000	0.000	0.000	0.152	0.087	0.000	0.000
		285	0.000	0.542	0.000	0.682	0.024	0.457	0.000	0.792	0.188	0.022	0.000	0.000	0.000	0.024	0.000	0.000	0.250	0.045	0.043	0.391	0.000	0.500
		291	0.000	0.000	0.000	0.000	0.000	0.000	0.000	0.000	0.000	0.000	0.000	0.000	0.000	0.000	0.017	0.000	0.000	0.000	0.000	0.000	0.000	0.354
		297	1.000	0.458	0.674	0.136	0.976	0.000	0.455	0.208	0.542	0.739	1.000	0.417	0.000	0.833	0.983	1.000	0.750	0.909	0.609	0.522	1.000	0.083
		300	0.000	0.000	0.000	0.045	0.000	0.500	0.341	0.000	0.000	0.000	0.000	0.000	0.000	0.024	0.000	0.000	0.000	0.000	0.196	0.000	0.000	0.063
		312	0.000	0.000	0.000	0.000	0.000	0.000	0.000	0.000	0.000	0.000	0.000	0.000	1.000	0.000	0.000	0.000	0.000	0.000	0.000	0.000	0.000	0.000
TB1019	Sample size		27	24	23	22	22	23	24	24	24	23	24	24	24	21	17	24	24	23	23	24	21	24
	Allele	272	0.074	0.979	0.283	0.409	0.045	0.457	0.917	0.417	0.146	0.413	0.750	0.208	0.229	0.833	0.559	0.875	0.708	0.913	0.696	0.354	0.595	0.375
		277	0.000	0.000	0.000	0.000	0.000	0.000	0.000	0.000	0.000	0.000	0.000	0.000	0.000	0.000	0.000	0.000	0.000	0.000	0.000	0.000	0.000	0.000
		283	0.926	0.021	0.391	0.523	0.955	0.543	0.000	0.583	0.438	0.543	0.250	0.792	0.771	0.048	0.412	0.125	0.292	0.087	0.152	0.479	0.405	0.625
		286	0.000	0.000	0.326	0.068	0.000	0.000	0.000	0.000	0.063	0.043	0.000	0.000	0.000	0.119	0.029	0.000	0.000	0.000	0.152	0.167	0.000	0.000
		289	0.000	0.000	0.000	0.000	0.000	0.000	0.000	0.000	0.000	0.000	0.000	0.000	0.000	0.000	0.000	0.000	0.000	0.000	0.000	0.000	0.000	0.000
		292	0.000	0.000	0.000	0.000	0.000	0.000	0.083	0.000	0.354	0.000	0.000	0.000	0.000	0.000	0.000	0.000	0.000	0.000	0.000	0.000	0.000	0.000
TB922	Sample size		32	24	19	21	18	20	17	23	24	20	22	23	23	21	29	23	24	23	23	20	13	17
	Allele	237	0.000	0.000	0.000	0.000	0.000	0.000	0.000	0.000	0.042	0.000	0.000	0.000	0.000	0.000	0.000	0.000	0.000	0.000	0.000	0.000	0.000	0.000
		239	0.000	0.000	0.000	0.476	0.000	0.200	0.118	0.587	0.167	0.350	0.000	0.000	0.000	0.000	0.914	0.000	0.000	0.000	0.000	0.000	0.000	0.000
		241	0.297	0.000	0.000	0.000	0.000	0.000	0.000	0.022	0.042	0.125	0.841	0.000	0.000	0.167	0.000	0.239	0.125	0.217	0.065	0.100	0.038	0.382
		243	0.000	0.000	0.000	0.000	0.000	0.025	0.000	0.000	0.042	0.000	0.159	0.000	0.348	0.024	0.052	0.000	0.000	0.000	0.022	0.000	0.000	0.000
		245	0.000	0.063	0.447	0.024	0.083	0.000	0.147	0.000	0.146	0.000	0.000	0.478	0.043	0.429	0.000	0.565	0.188	0.000	0.217	0.025	0.115	0.000
		247	0.000	0.000	0.500	0.381	0.000	0.225	0.000	0.000	0.021	0.000	0.000	0.000	0.000	0.119	0.017	0.000	0.146	0.000	0.348	0.100	0.692	0.000
		249	0.703	0.938	0.053	0.119	0.917	0.550	0.735	0.370	0.479	0.375	0.000	0.261	0.457	0.214	0.017	0.196	0.542	0.587	0.348	0.775	0.154	0.618
		251	0.000	0.000	0.000	0.000	0.000	0.000	0.000	0.022	0.063	0.150	0.000	0.000	0.152	0.024	0.000	0.000	0.000	0.196	0.000	0.000	0.000	0.000
		253	0.000	0.000	0.000	0.000	0.000	0.000	0.000	0.000	0.000	0.000	0.000	0.261	0.000	0.024	0.000	0.000	0.000	0.000	0.000	0.000	0.000	0.000

Green background = private allele
Yellow background = allele shared by two regions
Red number = fixed allele per locus
