# Supplementary material for: Multilocus genotype analysis outlines distinct histories for Trichinella britovi in the neighboring Mediterranean islands of Corsica and Sardinia
Source: Parasit Vectors. 2018 Jun 19;11:353. doi: 10.1186/s13071-018-2939-9 (PMC6006749; doi:10.1186/s13071-018-2939-9)
Supplement: Supplementary file 3 — Table S3. Pairwise Fst values of the 63 Trichinella britovi isolates. (PDF 240 kb) [file 13071_2018_2939_MOESM3_ESM.pdf]

Additional file 3: Table S3

Pairwise Fst values of the 63 *Trichinella britovi* isolates from the Corsica and Sardinia islands and from continental France, Italy and Spain based on four microsatellite markers. Not significant values ($P \geq 0.05$) are marked in blue. For the isolate numbers see Table 1.

SARDINIA; CORSICA (Cozzano, Vallée de Gravona, Bastelica, Aullène); ITALY; FRANCE; SPAIN

Isolate numb	1	2	3	4	5	6	7	8	9	10	11	12	13
1	0												
2	0.0407	0											
3	-0.02785	0.04957	0										
4	0.01045	0.0571	0.04729	0									
5	-0.05455	0.06711	-0.01571	0.06869	0								
6	-0.03759	0.10916	0.02409	0.13948	-0.00487	0							
7	-0.03437	0.08025	0.05103	0.13337	0.03363	-0.00081	0						
8	-0.04897	0.05281	-0.00131	0.0855	-0.00213	0.00197	0.00396	0					
9	-0.07601	0.05584	0.0093	0.06905	-0.00601	-0.01217	-0.00092	-0.00139	0				
10	-0.00834	0.12909	0.04992	0.15788	0.01794	-0.01481	0.00581	0.0149	0.02326	0			
11	-0.02865	0.08266	0.02561	0.06877	0.02504	0.0536	0.0777	0.04676	0.00261	0.12828	0		
12	-0.03324	0.12814	0.00947	0.09853	0.00028	0.00635	0.03949	0.00185	0.01843	0.01491	0.0664	0	
13	-0.02691	0.04323	0.02562	0.09612	0.03339	0.04112	0.01898	-0.00912	0.01746	0.06084	0.05943	0.04189	0
14	0.49562	0.49676	0.54301	0.54221	0.53938	0.54266	0.40686	0.4935	0.52251	0.51346	0.61898	0.57038	0.50649
15	0.476	0.45553	0.49742	0.54118	0.49017	0.46208	0.32092	0.42192	0.46668	0.41632	0.59508	0.50442	0.43279
16	0.49173	0.48831	0.53462	0.53927	0.53126	0.53017	0.39138	0.48163	0.5131	0.49769	0.61509	0.55882	0.4955
17	0.45347	0.46421	0.486	0.47055	0.48533	0.48709	0.38925	0.44747	0.46927	0.45811	0.5467	0.48747	0.47015
18	0.56026	0.55555	0.59656	0.57994	0.60038	0.62571	0.48806	0.5595	0.5987	0.6031	0.68185	0.6342	0.57208
19	0.38803	0.40407	0.44804	0.44958	0.43784	0.41966	0.31413	0.39223	0.40437	0.38836	0.49487	0.45028	0.40614
20	0.51131	0.50551	0.55292	0.55166	0.54962	0.55167	0.41565	0.50188	0.53076	0.52353	0.62659	0.57842	0.51253
21	0.38246	0.38924	0.43554	0.45095	0.42422	0.40086	0.27161	0.36757	0.38827	0.3633	0.50168	0.44184	0.3803
22	0.51311	0.49455	0.5372	0.56919	0.53259	0.51696	0.36462	0.46867	0.51309	0.4769	0.63537	0.55553	0.47903
23	0.57822	0.5459	0.58025	0.62089	0.58209	0.58298	0.41338	0.51369	0.58211	0.54187	0.7082	0.6094	0.52412
24	0.50561	0.49435	0.54345	0.5598	0.53804	0.5281	0.38195	0.48171	0.515	0.49446	0.62543	0.56617	0.4906
25	0.50453	0.48889	0.53366	0.5618	0.52827	0.51226	0.36252	0.46622	0.50619	0.4736	0.62581	0.5519	0.47628
26	0.51419	0.49699	0.53969	0.57156	0.53515	0.51931	0.36723	0.47147	0.51546	0.47962	0.63668	0.55819	0.48161
27	0.52684	0.50305	0.54319	0.57878	0.53988	0.52702	0.37065	0.47402	0.52487	0.48557	0.65037	0.56285	0.48482
28	0.53777	0.51474	0.55367	0.59071	0.55135	0.53973	0.38151	0.48508	0.53847	0.4986	0.66272	0.5748	0.49549
29	0.52676	0.50624	0.54673	0.58188	0.54336	0.52941	0.374	0.47805	0.52729	0.48871	0.65055	0.56636	0.48839
30	0.3945	0.33366	0.39716	0.38	0.38317	0.37776	0.2489	0.3375	0.34446	0.34496	0.4598	0.41075	0.35327
31	0.42252	0.42564	0.47449	0.46775	0.46596	0.45823	0.36122	0.42874	0.44047	0.42931	0.52109	0.48502	0.44322
32	0.52442	0.51264	0.56939	0.5562	0.56615	0.57856	0.46156	0.5282	0.55255	0.5567	0.62988	0.60151	0.53454
33	0.5119	0.52995	0.57094	0.56561	0.56525	0.57859	0.44758	0.52694	0.55302	0.55832	0.63964	0.60797	0.53535
34	0.38431	0.4315	0.45317	0.44153	0.44073	0.44013	0.33431	0.40586	0.41499	0.41867	0.49711	0.46361	0.42252
35	0.28186	0.31143	0.30729	0.33514	0.29942	0.27375	0.19663	0.25256	0.27944	0.23022	0.37873	0.27311	0.28478
36	0.25191	0.31084	0.25954	0.30006	0.24428	0.24782	0.23575	0.25113	0.2716	0.20239	0.34841	0.23795	0.30948
37	0.34659	0.38212	0.41954	0.4328	0.40644	0.37827	0.26549	0.35591	0.36657	0.34467	0.47018	0.41722	0.36979
38	0.19042	0.2405	0.21663	0.19217	0.22184	0.26809	0.24064	0.21657	0.24859	0.23935	0.29261	0.21729	0.24127
39	0.11208	0.24042	0.17067	0.24381	0.14636	0.12348	0.11248	0.1309	0.15666	0.08392	0.24025	0.11932	0.17655
40	0.37213	0.29818	0.37513	0.41269	0.38832	0.39875	0.27054	0.29128	0.38693	0.34181	0.50014	0.40053	0.26417
41	0.44056	0.34686	0.41646	0.45865	0.4395	0.47501	0.33895	0.33753	0.4619	0.41825	0.5739	0.45621	0.30505
42	0.61547	0.5768	0.6153	0.65196	0.61985	0.62938	0.44009	0.55017	0.62284	0.59319	0.74611	0.65574	0.55694
43	0.36222	0.40981	0.41025	0.40257	0.39312	0.41406	0.36599	0.40173	0.40605	0.38235	0.47326	0.43761	0.442
44	0.1768	0.28096	0.27466	0.3004	0.25628	0.22062	0.14935	0.21591	0.21741	0.19214	0.31197	0.23755	0.23734
45	0.33295	0.33655	0.33016	0.34094	0.32198	0.33377	0.29323	0.32165	0.3405	0.28433	0.42625	0.33708	0.37352
46	0.33422	0.45376	0.34533	0.49274	0.30364	0.25528	0.24791	0.27461	0.35507	0.19894	0.51534	0.28956	0.32414
47	0.47554	0.45403	0.46605	0.46629	0.46332	0.4733	0.41207	0.45011	0.47213	0.43361	0.54854	0.4748	0.48862
48	0.38178	0.41528	0.44214	0.41263	0.43513	0.43999	0.35992	0.41002	0.41545	0.41441	0.48051	0.44709	0.43002
49	0.50874	0.47122	0.49219	0.49927	0.48984	0.50716	0.43645	0.47884	0.50806	0.46124	0.59567	0.52031	0.51939
50	0.18566	0.22201	0.24911	0.22956	0.24312	0.2312	0.15531	0.20297	0.20791	0.18211	0.31286	0.22776	0.22206
51	0.14033	0.24073	0.2291	0.28076	0.20854	0.16212	0.09408	0.15824	0.17602	0.12153	0.28128	0.18727	0.18331
52	0.31586	0.45971	0.41222	0.46779	0.37929	0.38288	0.32464	0.36797	0.39361	0.36573	0.48548	0.42103	0.39537
53	0.33968	0.39217	0.36349	0.39779	0.36143	0.3379	0.25947	0.30713	0.34775	0.29804	0.44378	0.31196	0.3407
54	0.56942	0.49869	0.52257	0.52136	0.5448	0.59672	0.52107	0.50503	0.58356	0.56229	0.6452	0.55583	0.51334
55	0.51026	0.50806	0.55679	0.5416	0.55337	0.56114	0.43793	0.51363	0.53405	0.53815	0.61804	0.583	0.52313
56	0.37197	0.2912	0.39096	0.3933	0.38609	0.37907	0.21449	0.30397	0.34313	0.33797	0.47032	0.42183	0.28519
57	0.60092	0.58532	0.63481	0.62951	0.63733	0.66106	0.51031	0.5926	0.63518	0.64034	0.72352	0.68077	0.59791
58	0.25379	0.25386	0.31175	0.31556	0.30691	0.30988	0.22064	0.26199	0.29522	0.26277	0.38658	0.33484	0.273
59	0.39106	0.43516	0.46431	0.45401	0.45752	0.47223	0.35114	0.42571	0.45069	0.44033	0.54464	0.4925	0.45246
60	0.23352	0.31776	0.32706	0.33017	0.3076	0.28763	0.2187	0.28258	0.27661	0.26069	0.35497	0.31463	0.30742
61	0.28849	0.30794	0.33015	0.35044	0.31601	0.29628	0.22774	0.29051	0.29903	0.25066	0.39962	0.32693	0.32531
62	0.23883	0.31057	0.32556	0.37053	0.29975	0.26067	0.18326	0.25446	0.26618	0.22468	0.37192	0.31407	0.26826
63	0.25858	0.29316	0.25628	0.29548	0.24133	0.24644	0.23918	0.25153	0.27032	0.19663	0.34947	0.24639	0.3099

Isolate numb	14	15	16	17	18	19	20	21	22	23	24	25	26	27	28
14	0														
15	0.387	0													
16	0.00448	0.30279	0												
17	0.14118	0.40242	0.12498	0											
18	0.06293	0.55897	0.12559	0.09341	0										
19	0.00036	0.27684	0.01623	0.10913	0.04953	0									
20	-0.02144	0.36965	-0.01337	0.1614	0.06773	0.02303	0								
21	0.04269	0.18279	0.01536	0.15865	0.17997	0.01274	0.04076	0							
22	0.31266	0.03541	0.21336	0.3624	0.51382	0.23837	0.28885	0.13005	0						
23	0.57136	0.05553	0.48534	0.51936	0.73788	0.43147	0.55806	0.34915	0.12055	0					
24	0.09587	0.19252	0.03158	0.26478	0.29761	0.09906	0.07347	0.02341	0.09084	0.37531	0				
25	0.24478	0.06545	0.14954	0.3271	0.45075	0.19173	0.22005	0.08605	-0.01254	0.18592	0.03824	0			
26	0.3047	0.03908	0.20567	0.35992	0.50549	0.23434	0.28074	0.12522	-0.02159	0.12761	0.08398	-0.01437	0		
27	0.39791	0.01675	0.29881	0.40866	0.59096	0.29782	0.37645	0.19349	-0.00732	0.05114	0.17192	0.0254	-0.00367	0	
28	0.42306	0.01719	0.32475	0.42763	0.61089	0.31994	0.40248	0.21562	0.00272	0.03595	0.19744	0.04112	0.00709	-0.02119	0
29	0.37429	0.02107	0.27454	0.39884	0.5681	0.28407	0.35204	0.17632	-0.01424	0.07008	0.14734	0.01192	-0.01146	-0.02082	-0.01627
30	0.00391	0.28511	0.01916	0.13725	0.12811	-0.02812	0.06166	0.02883	0.26084	0.50434	0.12781	0.21037	0.25569	0.33128	0.35652
31	0.0676	0.37396	0.11179	0.16664	0.09776	0.01716	0.10572	0.1063	0.35018	0.52229	0.21227	0.30629	0.34688	0.40401	0.42481
32	0.18658	0.53862	0.26148	0.32806	0.21775	0.09612	0.21666	0.22767	0.52898	0.7082	0.35667	0.4787	0.5237	0.58909	0.6079
33	0.16413	0.57769	0.25823	0.35347	0.22909	0.13441	0.1996	0.21775	0.54968	0.7462	0.35138	0.4931	0.54266	0.61756	0.63598
34	0.13849	0.49426	0.20558	0.21138	0.13914	0.08628	0.17951	0.16559	0.46825	0.64172	0.31467	0.42164	0.46413	0.52447	0.54426
35	0.24525	0.211	0.19914	0.14063	0.30872	0.17788	0.25956	0.14533	0.2208	0.28784	0.24208	0.21752	0.22249	0.23134	0.24223
36	0.42574	0.44641	0.41458	0.33524	0.46735	0.34925	0.45104	0.35141	0.46828	0.52643	0.46307	0.46093	0.47042	0.47913	0.49123
37	0.029	0.20036	0.01098	0.12353	0.12807	0.01793	0.03353	-0.01743	0.14439	0.33576	0.04185	0.10429	0.14049	0.20023	0.22084
38	0.47844	0.52186	0.47913	0.38551	0.50071	0.3884	0.49652	0.40536	0.54289	0.60301	0.52298	0.53262	0.54496	0.55569	0.56849
39	0.40186	0.39105	0.39653	0.36211	0.46343	0.30995	0.42251	0.29823	0.42386	0.48532	0.41996	0.4164	0.42605	0.43523	0.44777
40	0.54619	0.41253	0.52157	0.5224	0.64003	0.42513	0.54504	0.39231	0.47523	0.54479	0.49653	0.47209	0.47635	0.48633	0.49646
41	0.68998	0.56953	0.67108	0.63691	0.76796	0.56123	0.69136	0.54976	0.63913	0.70626	0.65699	0.63594	0.63892	0.65108	0.6582
42	0.58146	0.02877	0.48703	0.54276	0.75624	0.44259	0.56874	0.35233	0.06386	-0.04642	0.35957	0.1394	0.0721	-0.01247	-0.02648
43	0.36505	0.58238	0.40404	0.3398	0.37673	0.29075	0.40726	0.35649	0.57738	0.68881	0.49107	0.54946	0.5761	0.61113	0.62618
44	0.2124	0.22355	0.18813	0.17046	0.28013	0.14439	0.22163	0.1073	0.22456	0.31662	0.21165	0.21208	0.22524	0.24508	0.25868
45	0.36439	0.41312	0.34004	0.22522	0.4017	0.29518	0.38773	0.30177	0.42088	0.49809	0.40725	0.4099	0.42231	0.43743	0.4508
46	0.80649	0.722	0.7971	0.73886	0.87835	0.67152	0.81466	0.67978	0.78792	0.87141	0.79318	0.78113	0.78488	0.80721	0.81056
47	0.4099	0.47696	0.38216	0.26769	0.44013	0.35127	0.43524	0.36724	0.47797	0.55996	0.46118	0.46588	0.47889	0.49637	0.50947
48	0.18955	0.48515	0.23479	0.16477	0.14848	0.13797	0.22861	0.21823	0.46308	0.59795	0.34979	0.4279	0.46137	0.50474	0.52294
49	0.44568	0.48465	0.40695	0.30475	0.50066	0.37874	0.46539	0.38712	0.48717	0.57924	0.47482	0.47487	0.48729	0.50809	0.52058
50	0.25285	0.21882	0.21858	0.18267	0.3121	0.18826	0.26032	0.14759	0.23169	0.2922	0.24242	0.22701	0.23359	0.24179	0.25288
51	0.19786	0.17955	0.17753	0.19342	0.28384	0.11672	0.21103	0.07724	0.19798	0.28956	0.18852	0.18619	0.19858	0.21814	0.23134
52	0.66597	0.71737	0.68325	0.64447	0.74343	0.52554	0.68435	0.56374	0.74496	0.83904	0.69955	0.72598	0.74218	0.77291	0.78131
53	0.38417	0.29023	0.33343	0.21077	0.44333	0.28646	0.38641	0.25969	0.31696	0.36185	0.36104	0.32057	0.31952	0.31917	0.32811
54	0.74076	0.72255	0.73188	0.66147	0.78064	0.64906	0.75016	0.66691	0.75395	0.80173	0.75201	0.7485	0.75405	0.76414	0.77063
55	0.01344	0.46434	0.06367	0.16014	-0.00317	0.04737	0.03158	0.11302	0.40814	0.63288	0.19891	0.34629	0.40153	0.48297	0.50619
56	0.19753	0.23289	0.17602	0.3193	0.34906	0.12527	0.20173	0.07784	0.22333	0.40555	0.15304	0.18974	0.21983	0.27423	0.293
57	0.04504	0.54993	0.11145	0.31214	0.1511	0.09958	0.04598	0.16129	0.49446	0.7612	0.22004	0.4168	0.48313	0.58899	0.61024
58	0.20003	0.34573	0.21265	0.23311	0.26111	0.13002	0.22252	0.13844	0.34061	0.47196	0.26061	0.31148	0.33954	0.37801	0.39589
59	0.08251	0.50105	0.16196	0.06726	0.08186	-0.01643	0.09986	0.12575	0.46738	0.66896	0.28206	0.41209	0.46139	0.53453	0.55525
60	0.11366	0.34088	0.13958	0.14024	0.13677	0.07631	0.1504	0.09663	0.31818	0.4564	0.22239	0.28558	0.31687	0.35866	0.37758
61	0.18369	0.20989	0.14324	0.13012	0.25411	0.1421	0.1994	0.10994	0.19925	0.29395	0.18875	0.18691	0.19967	0.22042	0.23367
62	0.30405	0.34484	0.3122	0.36988	0.41182	0.20339	0.31797	0.18979	0.36383	0.49298	0.30771	0.33929	0.36308	0.39813	0.41537
63	0.40278	0.41937	0.39312	0.32355	0.4509	0.32745	0.42986	0.33552	0.44178	0.49591	0.44008	0.43551	0.4442	0.45113	0.46314

Isolate numb	29	30	31	32	33	34	35	36	37	38	39	40	41
29	0												
30	0.31296	0											
31	0.39252	-0.04671	0										
32	0.57369	0.16645	0.06978	0									
33	0.59828	0.24612	0.16863	0.14044	0								
34	0.51141	0.17813	0.12196	0.15881	0.06329	0							
35	0.23193	0.14613	0.24088	0.40493	0.41752	0.29108	0						
36	0.48146	0.30121	0.36365	0.4975	0.50512	0.37831	0.14219	0					
37	0.1862	-0.00408	0.10508	0.21479	0.18268	0.12074	0.13337	0.33549	0				
38	0.55796	0.33781	0.39642	0.50049	0.51841	0.3833	0.24427	0.1569	0.37621	0			
39	0.4375	0.2721	0.33882	0.45172	0.44187	0.30993	0.15424	0.07275	0.28604	0.14967	0		
40	0.48589	0.43383	0.47174	0.58277	0.61715	0.53063	0.3369	0.45276	0.39238	0.39891	0.34989	0	
41	0.64808	0.59909	0.59576	0.70819	0.74528	0.65422	0.4419	0.5218	0.53491	0.45345	0.41799	0.02612	0
42	0.00828	0.55556	0.54105	0.72883	0.76404	0.66647	0.31821	0.5661	0.3284	0.63806	0.52514	0.58856	0.74799
43	0.60535	0.3047	0.27587	0.35402	0.32932	0.20284	0.35765	0.2814	0.32493	0.33214	0.29591	0.57136	0.66294
44	0.24313	0.08286	0.21376	0.33689	0.31967	0.19003	0.07473	0.23452	0.07863	0.22459	0.1738	0.33795	0.44385
45	0.43765	0.24747	0.31708	0.47114	0.49159	0.36884	0.13734	0.12027	0.2801	0.25227	0.24499	0.46593	0.55342
46	0.79771	0.75017	0.69953	0.81715	0.83574	0.72034	0.52487	0.47991	0.63809	0.52675	0.28692	0.63373	0.72404
47	0.49544	0.31037	0.36438	0.53092	0.56456	0.45511	0.15657	0.14201	0.34669	0.32625	0.31068	0.55314	0.64022
48	0.49755	0.1344	0.13196	0.21263	0.21832	0.09555	0.22748	0.2682	0.1896	0.28418	0.26718	0.50516	0.60939
49	0.50506	0.35665	0.40248	0.57018	0.60475	0.50325	0.24365	0.28392	0.36561	0.4354	0.41423	0.57872	0.67357
50	0.24323	0.10199	0.24257	0.36852	0.37965	0.26848	0.0657	0.21434	0.14322	0.21968	0.19997	0.25472	0.34792
51	0.21598	0.08736	0.18392	0.29685	0.28276	0.16422	0.06667	0.19779	0.07583	0.23997	0.10186	0.25431	0.36265
52	0.76432	0.6046	0.54242	0.62924	0.6024	0.45253	0.53234	0.47573	0.52079	0.4672	0.29015	0.63988	0.73443
53	0.32224	0.2709	0.36101	0.52378	0.53999	0.39726	0.03807	0.26152	0.23709	0.33871	0.24943	0.42277	0.51859
54	0.76272	0.66047	0.65322	0.75883	0.79184	0.70867	0.51692	0.50641	0.64592	0.45329	0.49682	0.55789	0.60871
55	0.4635	0.09178	0.09904	0.15394	0.14063	0.12433	0.30358	0.45876	0.07992	0.47116	0.43606	0.57332	0.70581
56	0.26069	0.20776	0.20463	0.30611	0.31013	0.28275	0.23387	0.41024	0.06706	0.38361	0.318	0.1779	0.36913
57	0.55899	0.23583	0.17357	0.22607	0.17599	0.23463	0.40849	0.55379	0.13631	0.58218	0.51952	0.65213	0.78878
58	0.37186	0.11209	0.15327	0.22753	0.23347	0.15734	0.19662	0.26572	0.14134	0.25097	0.21737	0.23839	0.36535
59	0.51694	-0.04519	-0.00896	0.09889	0.03707	-0.09836	0.2029	0.2818	0.07425	0.34186	0.27546	0.55497	0.68812
60	0.35201	0.04826	0.1054	0.18908	0.14601	0.03461	0.13607	0.19986	0.07238	0.2197	0.15841	0.40244	0.51333
61	0.21776	0.06902	0.19331	0.34073	0.35108	0.25312	0.06406	0.19263	0.09995	0.27795	0.23025	0.36554	0.47614
62	0.39241	0.20739	0.25107	0.31665	0.29	0.21335	0.26633	0.3331	0.18745	0.35255	0.20072	0.34549	0.46605
63	0.45413	0.23195	0.31901	0.47349	0.49311	0.37889	0.17666	0.07999	0.32446	0.21924	0.15673	0.43023	0.49767

Isolate numb	42	43	44	45	46	47	48	49	50	51	52	53	54	55
42	0													
43	0.71504	0												
44	0.32303	0.31644	0											
45	0.53481	0.25005	0.20993	0										
46	0.89904	0.68139	0.46655	0.60762	0									
47	0.59745	0.38866	0.30818	0.1036	0.71508	0								
48	0.6212	0.19407	0.22729	0.28875	0.68619	0.29192	0							
49	0.6155	0.3949	0.33854	0.05076	0.75941	0.15314	0.41351	0						
50	0.30027	0.32905	0.07963	0.15118	0.47847	0.24591	0.24762	0.24948	0					
51	0.30664	0.2743	0.03142	0.20639	0.37355	0.31017	0.21351	0.33446	0.08489	0				
52	0.86062	0.42922	0.42455	0.57932	0.65164	0.68361	0.49755	0.728	0.48182	0.33313	0			
53	0.40052	0.47321	0.12028	0.23344	0.58248	0.30596	0.36461	0.35485	0.12507	0.1223	0.61571	0		
54	0.83114	0.67271	0.55592	0.54524	0.8019	0.60681	0.62551	0.66724	0.47193	0.52263	0.78984	0.58524	0	
55	0.64844	0.36304	0.23156	0.39487	0.81777	0.44455	0.17243	0.48391	0.2854	0.24963	0.66921	0.42856	0.75037	0
56	0.43625	0.4241	0.14355	0.37886	0.6656	0.46475	0.29864	0.48013	0.11909	0.10178	0.56069	0.35266	0.63545	0.24721
57	0.77696	0.47741	0.34126	0.50974	0.90362	0.56352	0.29977	0.60058	0.37679	0.32539	0.769	0.53866	0.82433	0.05371
58	0.49581	0.19026	0.18083	0.22724	0.56707	0.33158	0.16926	0.33771	0.15185	0.113	0.40725	0.32246	0.5171	0.2255
59	0.68851	0.07265	0.17175	0.25373	0.77887	0.31355	-0.03258	0.39112	0.22587	0.14302	0.54628	0.36675	0.69398	0.01641
60	0.47434	0.15054	0.0796	0.21366	0.54201	0.26992	0.0479	0.34476	0.15958	0.07854	0.34716	0.25578	0.57857	0.12562
61	0.30294	0.2683	0.08279	0.06484	0.55496	0.16118	0.23317	0.11212	0.07581	0.09513	0.52403	0.15957	0.54838	0.22856
62	0.52299	0.31545	0.18629	0.36898	0.48385	0.46867	0.28805	0.49575	0.23844	0.09707	0.29772	0.36267	0.63054	0.33908
63	0.53599	0.25959	0.23794	0.07885	0.47644	0.19448	0.31206	0.20938	0.19863	0.19616	0.48198	0.26384	0.48522	0.44462

Isolate numb	56	57	58	59	60	61	62	63
56	0							
57	0.31285	0						
58	0.05137	0.29807	0					
59	0.2476	0.17316	0.10576	0				
60	0.16751	0.22726	0.10406	-0.06369	0			
61	0.17999	0.32818	0.15209	0.15687	0.12517	0		
62	0.17299	0.40116	0.15821	0.2409	0.1545	0.25167	0	
63	0.38781	0.53297	0.2435	0.27559	0.23449	0.15201	0.32595	0
